# Beyond Sacrificial Harm: A Two-Dimensional Model of Utilitarian Psychology

**DOI:** 10.1037/rev0000093

**Published:** 2017-12-21

**Authors:** Guy Kahane, Jim A. C. Everett, Brian D. Earp, Lucius Caviola, Nadira S. Faber, Molly J. Crockett, Julian Savulescu

**Affiliations:** 1 Uehiro Centre for Practical Ethics, University of Oxford; 2 Uehiro Centre for Practical Ethics and Department of Experimental Psychology, University of Oxford; 3 Uehiro Centre for Practical Ethics, University of Oxford; 4 Department of Experimental Psychology, University of Oxford; 5 Uehiro Centre for Practical Ethics and Department of Experimental Psychology, University of Oxford; 6 Department of Experimental Psychology, University of Oxford; 7 Uehiro Centre for Practical Ethics, University of Oxford

**Keywords:** empathy, impartiality, moral dilemmas, moral psychology, utilitarianism

## Abstract

Recent research has relied on trolley-type sacrificial moral dilemmas to study utilitarian versus nonutilitarian modes of moral decision-making. This research has generated important insights into people’s attitudes toward instrumental harm—that is, the sacrifice of an individual to save a greater number. But this approach also has serious limitations. Most notably, it ignores the positive, altruistic core of utilitarianism, which is characterized by impartial concern for the well-being of everyone, whether near or far. Here, we develop, refine, and validate a new scale—the Oxford Utilitarianism Scale—to dissociate individual differences in the ‘negative’ (permissive attitude toward instrumental harm) and ‘positive’ (impartial concern for the greater good) dimensions of utilitarian thinking as manifested in the general population. We show that these are two independent dimensions of proto-utilitarian tendencies in the lay population, each exhibiting a distinct psychological profile. Empathic concern, identification with the whole of humanity, and concern for future generations were positively associated with impartial beneficence but negatively associated with instrumental harm; and although instrumental harm was associated with subclinical psychopathy, impartial beneficence was associated with higher religiosity. Importantly, although these two dimensions were independent in the lay population, they were closely associated in a sample of moral philosophers. Acknowledging this dissociation between the instrumental harm and impartial beneficence components of utilitarian thinking in ordinary people can clarify existing debates about the nature of moral psychology and its relation to moral philosophy as well as generate fruitful avenues for further research.

According to classical utilitarianism, we should always act in the way that would maximize aggregate well-being. Since its introduction in the 18th century by the philosopher Jeremy Bentham, this simple idea has been massively influential—and massively controversial. Modern-day secular morality can be seen as the gradual expansion of our circle of moral concern from those who are emotionally close, physically near, or similar to us, to cover the whole of humanity, and even all sentient life (Singer, 1981; see also Pinker, 2011). Utilitarians like Bentham, John Stuart Mill, and, in our time, Peter Singer, have played a pivotal role in this process, and in progressive causes more generally. They have been leading figures in the fights against sexism, racism, and ‘speciesism;’ influential supporters of political and sexual liberty; and key actors in attempts to eradicate poverty in developing countries as well as to encourage more permissive attitudes to prenatal screening, abortion, and euthanasia within our own societies (Bentham, 1789/1983; Mill, 1863; Singer, 2011). Yet utilitarians have never constituted more than a tiny minority, and utilitarianism has always faced fierce resistance. Pope John Paul II famously wrote: “Utilitarianism is a civilization of production and of use, a civilization of ‘things’ and not of ‘persons,’ a civilization in which persons are used in the same way as things are used” (John Paul II, 1995). But it is not only defenders of traditional morality who reject utilitarianism; prominent progressive thinkers have criticized utilitarianism in similar terms (Rawls, 1971; Williams, 1973), and many continue to angrily protest the views of utilitarians such as Singer (Schaler, 2009). Clearly, utilitarianism is a distinctive, influential, and controversial ethical view.

Given the influential but controversial reach of utilitarianism in ethics and society, questions about the psychological basis of utilitarian moral thinking—and why some people are so attracted to it while others are so repelled—have been of considerable interest to philosophers and psychologists alike. Utilitarians have often answered such questions by appealing to a contrast between cool logic and misguided intuitions and emotions. They argue that common moral views have their source in gut reactions and intuitions shaped by discredited religious views or evolutionary pressures, and that careful reflection should lead us to abandon these views and endorse utilitarianism, a more logical view based in rational reflection (Singer, 2005). Recognizing that this notion is, in part, a testable hypothesis about human moral psychology, some advocates of utilitarianism have generated an influential body of empirical research that has by and large seemed to confirm it.

The main approach in this research has been to study responses to ‘sacrificial’ moral dilemmas (such as the famous ‘trolley’ scenario and its various permutations; see Foot, 1967) which present a choice between sacrificing one innocent person to save a greater number of people, or doing nothing and letting them die. In analyzing these responses and relating them to other variables, such as individual difference scores on personality measures or patterns of brain activity, researchers have tried to uncover the psychological and even neural underpinnings of the dispute between utilitarians and their opponents—such as defenders of deontological, rights-based views of the kind associated with Immanuel Kant.

In keeping with the ‘cool logic’ versus ‘misguided emotions’ framework, these researchers have made heavy use of a dual-process approach to understanding human cognition. Dual process models conceptualize cognition as resulting from the competition between quick, intuitive, and automatic processes, and slow, deliberative, and controlled processes (e.g., Bargh & Chartrand, 1999; Chaiken & Trope, 1999; Shiffrin & Schneider, 1977). Running with this idea, influential research by Greene and colleagues has applied a dual process lens to our moral judgments to suggest that while deontological judgments (refusing to sacrifice the one) are based in immediate intuition and emotional gutreactions, utilitarian judgments (sacrificing one to save a greater number) are uniquely attributable to effortful reasoning (Greene, 2007; Paxton, Bruni, & Greene, 2014). It has also been suggested that these opposing utilitarian and deontological forms of decision-making are based in distinct neural systems (Greene, Nystrom, Engell, Darley, & Cohen, 2004).

Despite an abundance of early findings in support of Greene’s account, more recent research has yielded results that are more difficult to square with its—ultimately flattering—picture of utilitarian thinking. For example, multiple studies have reported an association between ‘utilitarian’ responses to sacrificial dilemmas and psychopathy and, more generally, aggressive and antisocial tendencies including reduced concern about harm to others (Bartels & Pizarro, 2011; Glenn, Koleva, Iyer, Graham, & Ditto, 2010; Kahane, Everett, Earp, Farias, & Savulescu, 2015; Wiech et al., 2013). These findings are puzzling. Utilitarians are supposed to care about the good of all sentient beings; psychopaths notoriously care only about their own good. So why is psychopathy one of the traits most consistently associated with what are supposed to be paradigm cases of utilitarian judgment?

The answer to this puzzle may be found by way of illustration. In March 2017, disability activists outraged by Peter Singer’s support for the infanticide of severely disabled babies prevented him from speaking (via an Internet link) at an event organized by the Effective Altruism Club of Victoria University in Canada—a club whose founding, in turn, was inspired by Singer’s advocacy of self-sacrifice in the name of charity (Singer, 2015). This incident—and the two ‘sides’ of Singer’s views attracting both censure and praise—offers the beginnings of an answer to our question by showing two distinct ways in which utilitarianism radically departs from commonsense morality.

The first way utilitarianism departs from such commonsense morality is that it places no constraints whatsoever on the maximization of aggregate well-being. If killing a severely disabled child would lead to more good overall—as Singer believes is at least sometimes the case—then utilitarianism, in stark contrast to commonsense morality, requires that the child be killed. This explains the angry protests at Singer’s talk. But this requirement is just one aspect of utilitarianism: specifically, it is the *negative* dimension according to which we are permitted (and even required) to instrumentally use, severely harm, or even kill innocent people to promote the greater good. We call this dimension ‘instrumental harm.’

There is also a positive dimension to utilitarianism, and this dimension, too, departs from commonsense morality. Recall that utilitarianism requires us to maximize, not our own preferences or well-being—not even that of those near or dear to us, or of our compatriots—but the well-being of all sentient beings on the planet, and to do so in such a way that “[e]ach is to count for one and none for more than one” (Bentham, 1789/1983). This dimension explains why utilitarianism is sometimes described as a form of universal or impartial beneficence (which is what we shall call this positive dimension). For people in affluent countries, the demand to impartially maximize welfare is likely to require significant self-sacrifice—for example, giving much of our income to charity. And although many find this level of sacrifice far too demanding (Cullity, 2006), this impartial ideal has inspired a global movement of ‘effective altruists,’ including those in attendance at Singer’s event at Victoria University (MacAskill, 2015; Singer, 2015).

Now we can resolve the puzzle. The sacrificial dilemmas paradigm, we claim, has yielded such strange and even contradictory findings because it focuses almost exclusively on the negative side of utilitarian decision-making. So, while psychopaths may be more willing to push someone off a footbridge to save five others (or be less shocked by support for infanticide or euthanasia), it would be surprising if these same psychopaths signed up to join an Effective Altruism Club or showed care for the plight of strangers in the developing world. In other words, the sacrificial dilemmas paradigm ignores or downplays the positive, impartial and altruistic core of a utilitarian approach to ethics. Accordingly, over a decade of research employing sacrificial dilemmas to study ‘utilitarian’ thinking has shed light only or primarily on instrumental harm: the conditions under which people find it acceptable to cause harm for a greater good. Such dilemmas, however, tell us little about the sources of impartial concern for the greater good, despite the fact that this positive, all-encompassing altruistic aim is at the very heart of a utilitarian approach. In short, recent research has told only half of the story about the psychology of utilitarianism. And because impartial beneficence is the philosophical core of utilitarian thought—whereas acceptance of instrumental harm is one implication of that central core, when it is endorsed without qualification—it has arguably focused on the less important half.

Our paper has three aims. First, we will propose a new conceptual framework for thinking about the psychology of utilitarian tendencies in the lay population. We hope that this framework will also serve as a general model for thinking about the relationship between the explicit ethical theories debated by philosophers and the pretheoretical moral decision-making of ordinary people. Second, using this framework, we will outline a new approach for studying individual differences in proto-utilitarian tendencies. We will introduce and validate a new scale—the Oxford Utilitarianism Scale (OUS)—that was designed to address important limitations of the sacrificial dilemmas paradigm. Third, we will propose a new theory of the psychological sources of proto-utilitarian modes of thinking, a theory that can explain the recent (puzzling) findings about utilitarian judgment as well as generate new directions for future research.

Current work in moral psychology has largely assumed that utilitarian decision-making is a unitary psychological phenomenon. By contrast, the *Two Dimensional* (2D) model of utilitarian thinking we develop here highlights the distinct positive and negative components of utilitarian decision-making. Although these two dimensions overlap in explicit utilitarian theorizing, they often come apart in the moral thinking of lay persons and are indeed in some tension in that domain. We will end by exploring the theoretical, methodological, and practical implications of this overlooked division within utilitarian thinking. We will highlight, in particular, the way in which the 2D model casts doubt upon dominant philosophical and psychological accounts both of the psychological basis of utilitarianism and of the sources of continuing resistance to it.

## Utilitarianism and Moral Decision-Making in the Lay Population

### Ethical Theory and Moral Judgment in Nonphilosophers

Although there is a large and growing body of psychological research into utilitarian decision-making, this research has largely proceeded without a precise account of the sense in which the moral judgments of nonphilosophers can usefully be described be in terms drawn from explicit philosophical theories (but see Greene, 2008).

Moral philosophers develop, elaborate, and debate explicit ethical theories. But although some ethical theories, or ideas derived from such theories (e.g., the Kantian concept of human dignity), occasionally become more widely known, there is little reason to think that lay people employ explicit ethical theories in forming their moral judgments—let alone the specific theories debated by academic philosophers. It is plausible, however, that such philosophical theories draw upon pretheoretical moral intuitions and tendencies. It is also likely that both attraction to, and rejection of, explicit ethical theories is driven, at least in part, by individual differences in such pretheoretical moral tendencies. Such tendencies would involve being responsive to and emphasizing the factors that a given ethical theory regards as morally relevant. They will therefore often also be reflected by patterns of moral judgments that at least partly mirror those supported by the theory. Less centrally, such tendencies can also involve forms of moral reasoning and deliberation that echo (or are the precursors of) those recommended by the theory.

Importantly, we should not treat such pretheoretical tendencies in an all-or-nothing manner; few if any nonphilosophers are full-blown utilitarians. Such tendencies are rather a matter of degree: the moral thinking of ordinary individuals will approximate to a greater or lesser extent—as well as in some but not other respects—the patterns of judgments and response that characterize a given explicit ethical theory.

One could still ask, however: Even if nonphilosophers do not form their moral judgments by applying an explicit ethical theory, why not simply ask them to what extent they endorse such a theory? Although tempting, this approach is not, we claim, a promising way to measure the moral views of ordinary people. To begin with, there is considerable evidence that people often do not have introspective access to the principles and factors to which their moral judgments are actually responsive. In addition, their judgments about concrete cases needn’t reflect the general moral principles that they would endorse upon reflection (Cushman, Young, & Hauser, 2006; Hauser, Cushman, Young, Kang-Xing Jin, & Mikhail, 2007; Lombrozo, 2009). To illustrate: the utilitarian idea that we should act in ways that promote everyone’s happiness can sound very attractive in the abstract, but many reject utilitarianism when they realize the highly counterintuitive implications of treating this idea as the sole criterion of moral action—that is, its uncompromising demand for impartiality and self-sacrifice, on the one hand, and for the sacrifice of innocent others for the greater good, on the other (as, e.g., in Judith Jarvis Thomson’s [Thomson, 1985] case of killing a patient and using his organs to save five others; see Horne, Powell, & Hummel, 2015). Thus, even when people endorse the core utilitarian principle in the abstract, their actual moral judgments may still be guided by deontological considerations relating to rights, duties, or degrees of personal relationship. In fact, lay people who endorse utilitarian principles in the abstract do not tend to also reject opposing deontological principles (Tanner, Medin, & Iliev, 2008). Therefore, to measure the extent to which people approximate an ethical theory such as utilitarianism, we need to approach things more indirectly, by examining a broader range of patterns of moral thought and judgment (Kahane & Shackel, 2010).

Finally, we need to strike a balance between philosophical accuracy and empirical plausibility. It is unlikely that the moral judgments of nonphilosophers mirror the most intricate and subtle forms of the ethical theories developed by philosophers, nor should we expect the moral views of ordinary people to be fully consistent. At the same time, if we use terms such as ‘utilitarian’ too loosely, these terms will lack any interesting theoretical content and, indeed, will mislead us into reading more into more mundane forms of ordinary moral judgment than is really there (Kahane, 2015). In the next section, we give specific examples to illustrate these considerations.

### Understanding Utilitarianism

Utilitarianism involves more than the commonplace ideas that we should aim to prevent suffering and promote happiness, or that it is morally better to save more rather than fewer lives (all else equal). Utilitarians make a far more radical claim: that we should adopt a thoroughly impartial standpoint, aiming to maximize the well-being of all persons (or even all sentient beings), regardless of personal, emotional, spatial, or temporal distance (*positive dimension*); and that this should be our one and only aim, unconstrained by any other moral rules, including rules forbidding us from intentionally harming innocent others (*negative dimension*). In line with the framework set out above, it is worth pausing to spell out the distinctive patterns of moral thought and judgment involved in each of these dimensions of utilitarianism.

#### The positive dimension of utilitarianism: Impartiality

The philosophical core of utilitarianism lies in the impartial maximization of the greater good.^1^ To adopt a thoroughly impartial moral standpoint is to treat the well-being of every individual as equally important. No priority should be given to one’s own good, nor to that of one’s family, friends, compatriots, or even fellow humans over nonhuman animals. Such a standpoint would normally imply highly demanding forms of self-sacrifice—whether by becoming vegetarian or vegan, giving much of one’s money to effective charities aiming to relieve suffering in distant countries, or perhaps even donating one’s own kidney. Indeed, utilitarianism instructs moral agents to sacrifice their own well-being even if there is only a tiny increment in the well-being of others over what they themselves have lost.

Notice, however, that such moral impartiality is not the same thing as altruism and self-sacrifice. Someone might not hesitate to risk their life to save a drowning child, while at the same time failing to conclude that they have any reason to give up an affluent lifestyle. Ordinary, ‘commonsense’ morality encourages modest acts of altruism (e.g., helping a beggar or making an occasional donation to charity) and rewards heroism in the context of acute emergencies. But complete impartiality requires more—much more. The utilitarian Peter Singer, for example, is as we noted a leading proponent of effective altruism, a movement built around the idea of using reason and evidence to identify the best ways of helping others. Many effective altruists have pledged to give at least 10% of their income to cost-effective charities (MacAskill, 2015; Singer, 2015)—and even this arguably falls considerably short of the strict utilitarian ideal. In the U.K., the median amount given to charity per year is £168, and of the four most popular causes that people donate to (charities focused on children, medical research, animals, and hospices: Charities Aid Foundation, 2016), none are focused on the developing world, where arguably the most good can be done. With a median salary of around £27,000, on utilitarian effective altruism principles we should donate at least £2,700—or 16 times the actual amount—and we should send it to charities that would impartially do the most good. In fact, people typically do neither.

Utilitarianism diverges from ordinary morality not only with respect to how *much* we should sacrifice but also for *whose* sake. Some individuals engage in acts of extreme self-sacrifice—in some cases, sacrificing their lives to promote the good of their family, country, or religious group. But such altruistic acts are hardly expressions of impartiality since they focus on one’s friends, family, or ingroup. Utilitarianism, by contrast, actually forbids us from giving any special priority to those close to us over others (saving the lives of compatriots, or even family, before those of distant strangers); indeed, if it would maximize welfare, we must make sacrifices for our greatest enemies. Of course, how much one approximates this impartial ideal is a matter of degree—even avowed utilitarians admit that they fail to realize it without qualification (Singer, 2015). Finally, there is more than one way of departing from this ideal: a religious fundamentalist may discount self interest in favor of her ingroup while an egoist might care only about himself without differentiating much between strangers and his closest family members.^2^

#### The negative dimension of utilitarianism: Harming and breaking rules

Although a thoroughly impartial moral outlook is necessary for utilitarianism, it is not sufficient. One can adopt such an outlook while still holding that the goal of maximizing everyone’s well-being must only be pursued in line with various moral rules constraining us from certain ways of harming innocent people, lying, breaking promises, and the like. In other words, even if one endorses this impartial moral *goal*, one may still think that we are forbidden from taking certain *means* to achieve it. The negative component of classical utilitarianism is the denial that there are any such constraints. We should of course still usually tell the truth, keep our promises, and refuse to harm innocent people—but only when (and because) these acts are likely to lead to a better impartial outcome. When they get in the way of achieving such an outcome, such familiar moral rules can and should be broken.

The most central of these rules relates to what we called instrumental harm—willingness to harm and even kill others when this is needed to achieve a better outcome. Such a willingness can be seen when—as in the classic thought experiment—someone pushes an innocent person off a footbridge to save a greater number of lives. But it can also be seen in more realistic examples, such as when someone holds that torture is morally acceptable if needed to reduce the risk of a major terrorist attack. Similar reasoning explains why some utilitarians support the legalization of so-called ‘active euthanasia’ as well as, more controversially, the abortion (and in some cases even infanticide) of the severely disabled. However, willingness to cause instrumental harm is not the only way in which utilitarians reject the authority of many other putative moral rules—including, as mentioned, those relating to honesty and keeping promises, as well as to fairness, hierarchy, and ‘purity’ (i.e., a concern for strict sexual and other boundaries). It is for this reason that utilitarians were among the earliest to support the legalization of homosexuality and, more generally, to defend a permissive attitude toward sexuality (Bentham, 1785/1978).

One final clarification. Just as one can endorse an impartial aim to maximize welfare without rejecting common moral constraints, so can one reject many or even most of these rules and values without endorsing the impartial positive aim of utilitarianism. An avowed egoist, for example, might also regard constraints against lying or even killing in a purely instrumental way yet see no reason at all to care about the greater good.^3^

### Degrees of Proto-Utilitarian Tendencies in Lay Moral Judgment

Understood as an explicit ethical theory, classical utilitarianism is firmly committed both to unqualified impartiality and to the rejection of all inherent moral constraints on the maximization of aggregate well-being. Now, few if any nonphilosophers are likely to consciously apply such an explicit theory. However, the moral thinking of ordinary people may approximate such an outlook to varying degrees. We propose that the closer a person approaches moral questions in ways that give weight to the concepts and considerations central to paradigmatic utilitarianism (i.e., the ‘classic’ view associated with Bentham and Mill), the stronger the utilitarian tendencies of that individual. Spelled out in the terms set out above, a person’s moral thinking should count as more utilitarian (a) the greater its focus on the impartial maximization of well-being across different moral contexts (*positive dimension*) and (b) the less space and weight it gives to values other than well-being, and to moral rules constraining the promotion of well-being (*negative dimension*).^4^

With respect to (a), we have seen that an individual can reject impartial morality both by privileging the self and by privileging family, friends, or compatriots, or generally those who are spatially and temporally closer. With respect to (b), the other values and rules in question could be both a matter of *number* (e.g., traditional morality accepts multiple moral rules that can constrain the promotion of aggregate well-being, such as rules relating to hierarchy, purity, and so on; see Haidt, 2012) and *strength* (e.g., libertarians typically accept far fewer moral rules than traditionalists, but the few rules they do accept with respect to, e.g., property rights are extremely strong). In their strongest form, such competing moral rules state absolute prohibitions. But they needn’t be as strong as that. Many nonutilitarians accept that we can break certain moral rules (e.g., relating to truth-telling or promises) when adhering to them would lead to significant harm (Holyoak & Powell, 2016), and plenty of nonutilitarians are willing to endorse causing severe harm to innocents in situations where the costs of refusing to do so are catastrophic (Fried, 1978). Stronger rules will have higher thresholds, and different individuals will draw those thresholds at different points (Trémolière & Bonnefon, 2014).

Someone who thinks about morality in unqualifiedly impartial terms, privileging no one over another while rejecting any constraint whatsoever on the maximization of well-being, would count as fully utilitarian on the proposed construct. Such a person would closely conform, at least in their moral thinking, to classical act utilitarianism, and to the form of utilitarianism presently defended by philosophers such as Peter Singer (2011).

Utilitarianism can also take other forms. One is ‘rule’ utilitarianism, which holds that the morally right action is the one that conforms to rules that, if widely adhered to, would maximize well-being. There are also nonutilitarian forms of consequentialism that recognize values beyond that of utility (e.g., the value of fairness) and even accommodate forms of partiality (Scheffler, 1982; Sen, 1983). Because they depart from classical utilitarianism in ways that bring them closer to commonsense morality, adherents of such views would count as somewhat less utilitarian on the proposed construct.

It may be worth clarifying at the outset why we privilege classical act utilitarianism in this way. First, as a minor point, this is the form of utilitarianism assumed by most work in current moral psychology; our framework aims to improve on existing practice but also to be continuous with it rather than to change the subject. Second and more importantly, classical act utilitarianism is the original form of the view and remains the most famous, most influential, and most controversial. Third, some more recent developments of utilitarianism (e.g., ‘motive’ or ‘global’ utilitarianism, or the distinction between criterion-of-rightness and decision-procedure; see Sinnott-Armstrong, 2015) are probably too subtle or complex to be reflected in the moral thinking of nonphilosophers: it is not by accident that it took many decades of intense philosophical reflection to identify these variants. Fourth, most important deviations from classical act utilitarianism—rule utilitarianism, satisficing utilitarianism and nonutilitarian consequentialism being prime examples—are attempts to bring utilitarianism closer to commonsense morality and tone down its more radical and counterintuitive aspects. Since we are proposing a way to rank moral outlooks as *more* or *less* utilitarian, it is hard to see what could replace the most unqualified form of the view (which also happens to be the most paradigmatic) at the ‘top’ of the scale. Importantly, however, other forms of utilitarianism are not ignored by our framework—their adherents would be ranked as leaning strongly toward utilitarianism but somewhat less so than classical utilitarians. This seems exactly right.

As we move further away from utilitarianism in its paradigmatic forms, we find approaches to morality that are increasingly partial and that recognize a greater number of increasingly stringent deontological constraints. Individuals who think of morality in these ways would count as low on utilitarian dispositions. Notice, though, that the construct we are developing is a measure of utilitarian tendencies, not a general taxonomy of possible moral views. There are multiple ways to reject utilitarianism—we already mentioned traditional morality, libertarianism, Kantian ethics and other rights-based approaches; there is also virtue ethics (Hursthouse, 1999) as well as others. These are very different nonutilitarian views, and the proposed construct is not intended to differentiate between them.

Finally, although we explained above what would count as a stronger or weaker utilitarian tendency by reference to a range of utilitarian and nonutilitarian theories, it bears emphasizing again that the construct we have in mind is a measure of broad tendencies in moral deliberation and judgment in the lay population. It is not likely that ordinary people apply anything resembling an explicit ethical theory (whether utilitarian or not), nor is it likely that their moral judgments are fully consistent across different moral contexts.

## Existing Measures of Utilitarian Decision-Making

### Sacrificial Dilemmas

Armed with this theoretical framework, we can return to the sacrificial dilemmas paradigm. Sacrificial dilemmas are by far the most dominant experimental paradigm in contemporary moral psychology (Christensen & Gomila, 2012), and are widely assumed to be a reliable measure of utilitarian decision-making. Prosacrifice responses to such dilemmas are routinely classified as ‘utilitarian judgments,’ and the psychological processes and mechanisms implicated in such judgments interpreted as reflecting general features of utilitarian decision-making (Greene, 2008). Moreover, the number of prosacrifice responses to batteries of such dilemmas are widely used as measures of differences in utilitarian tendencies both within (Bègue & Laine, 2017; Lombrozo, 2009) and between populations (Koenigs et al., 2007).

The work of Greene and colleagues on sacrificial dilemmas has been deeply influential to the field. It has spurred more than a decade of fascinating research in moral psychology, and has made substantial advances to our understanding of instrumental harm. That said, despite—or perhaps because of—how popular sacrificial dilemmas have been in moral psychology, this approach has naturally invited some criticism, for example, relating to the highly artificial character of the scenarios typically used (Bauman, McGraw, Bartels, & Warren, 2014). Our aim here is not to offer further criticism of this paradigm but to highlight its limits as a general measure of utilitarian tendencies.

Sacrificial dilemmas only directly measure what we call the negative dimension of utilitarianism. In fact, they measure only attitudes to instrumental harm—just one aspect of the negative dimension, albeit a very important one, given the moral centrality of prohibitions against harming others. To judge that, for example, we should push one innocent person off a footbridge is to reject (or at least discount) one possible deontological rule against directly harming someone (as a means to preventing a greater harm to others). But one could reject this particular deontological rule while still accepting many other rules—for example, rules relating to fairness, honesty, or promise-keeping. And one can certainly reject this rule while remaining highly partial in one’s moral decision-making.

That someone makes a judgment that happens to be in line with utilitarianism in a specific context does not, of course, immediately show that their judgments stem from, or are responsive to, the considerations that lie at the heart of a utilitarian moral outlook. Nor does it show that they will make judgments in a way that resembles such an outlook in other contexts. It is nevertheless an empirical possibility that prosacrifice responses to sacrificial dilemmas reflect the application of a general utilitarian principle (‘impartially maximize expected utility’) or even indicate a broader tendency to approach moral decision-making in a utilitarian manner. If that were the case, this could potentially justify the near exclusive focus on sacrificial dilemmas, because utilitarian decision-making would be a *unitary* psychological phenomenon in the context of the lay population, with a common set of processes and factors leading to judgments in line with utilitarianism in a range of moral contexts. By studying responses to sacrificial dilemmas, we would be shedding light on this general phenomenon. This assumption is implicit in Greene’s pioneering research (Greene, 2008), which is largely focused on sacrificial dilemmas yet makes general claims about utilitarian decision-making and judgments. But the assumption that utilitarian decision-making is a unitary psychological phenomenon that is fully (or even uniquely) reflected in prosacrifice responses to sacrificial dilemmas also seems to underlie much other research employing such dilemmas whereby results are routinely stated as supporting *general* conclusions about the psychology of utilitarian decision-making (see, e.g., Côté, Piff, & Willer, 2013; Duke & Bègue, 2015; Koenigs et al., 2007; Robinson, Joel, & Plaks, 2015). And yet, the association we discussed earlier between ‘utilitarian’ judgments in sacrificial dilemmas and antisocial traits such as psychopathy in both clinical (Koenigs et al., 2012) and subclinical (Bartels & Pizarro, 2011; Glenn et al., 2010; Kahane et al., 2015; Wiech et al., 2013) populations casts doubt upon this assumption. Still other studies have shown a relationship between prosacrifice judgments and libertarian political views (Iyer, Koleva, Graham, Ditto, & Haidt, 2012) as well as explicit endorsement of various forms of egoism (Kahane et al., 2015), both of which give heightened or even exclusive priority to one’s own self-interest over the welfare of others.

Psychopaths are obviously not paragons of impartial concern for the greater good, and egoists explicitly reject any such concern. These findings directly contradict the strict impartial concern for all people’s interests that is at the core of utilitarian theory. Further departure from such impartiality is seen in research suggesting that prosacrifice judgments are more likely to be made when they are in the participants’ self-interest (Moore, Clark, & Kane, 2008), and in research suggesting that rates of prosacrifice judgments are strongly influenced by in-group membership: whether the comparison is between foreigners versus compatriots (Swann, Gomez, Dovidio, Hart, & Jetten, 2010), strangers versus family members (Petrinovich, O’Neill, & Jorgensen, 1993), or even animals versus humans (Petrinovich et al., 1993), in-group members are more likely to be saved. Such findings suggest that prosacrifice judgments in these dilemmas are rarely based in the kind of impartial maximization of aggregate welfare that utilitarianism demands.

In Kahane et al. (2015) we directly investigated the relationship between a tendency to make prosacrifice judgments in sacrificial dilemmas and a wide range of measures of impartial moral concern for the greater good in other contexts. Such measures included: willingness to donate money to reduce the suffering of those in need in poor countries, rejecting favoritism toward one’s compatriots over distant strangers, and identifying with the whole of humanity. We consistently found either no relation or a negative relation between prosacrifice judgments and such impartial concern for the greater good (Kahane et al., 2015). These findings suggest that an impartial moral view may actually be in tension with a permissive attitude toward instrumental harm. Our hypothesis gains some support from other recent research. Although psychopathy has been associated with smaller amygdalae, reduced amygdala responses to fear-related stimuli, inferior ability to recognize fearful expressions, (Dawel, O’Kearney, McKone, & Palermo, 2012; Marsh & Blair, 2008), and reduced empathic concern (Decety, Lewis, & Cowell, 2015), recent studies of individuals who donated their kidney to a complete stranger—an extreme form of altruism which is strongly consistent with the positive core of utilitarianism—found that such individuals have large right amygdalae and superior ability to recognize fearful expressions, compared with control subjects (Marsh et al., 2014). Such extreme altruism was also found to be associated with higher empathic concern (Brethel-Haurwitz, Stoycos, Cardinale, Huebner, & Marsh, 2016) and reduced social discounting toward distant strangers (Vekaria, Brethel-Haurwitz, Cardinale, Stoycos, & Marsh, 2017), indicating greater impartiality.

Utilitarian judgments in sacrificial dilemmas clearly do not measure the positive dimension of utilitarianism. In fact, it is not clear that they measure its negative dimension more generally, beyond attitudes to instrumental harm. For example, Kahane et al. (2012) found no association between prosacrifice judgments in sacrificial dilemmas and greater endorsement of lying when it leads to greater overall utility (another characteristic utilitarian judgment). It thus seems that rejecting moral rules relating to instrumental harm does not predict the rejection of deontological rules in other domains (e.g., relating to honesty).

These results make sense when one considers that although utilitarians and deontologists often do endorse contrasting responses to sacrificial dilemmas, these dilemmas were not designed by philosophers as a way of bringing out the core disagreement between utilitarianism and its opponents. In fact—contrary to what is commonly assumed by many researchers—the ‘trolley’ scenarios on which sacrificial dilemmas are based were actually introduced as a way of exploring certain issues *within* the deontological approach (see Foot, 1967; Thomson, 1985). Thus, although sacrificial dilemmas were an important first step in studying utilitarian decision-making, and have already yielded valuable findings about attitudes in favor of and against instrumental harm, they need to be supplemented with further tools that allow us to study utilitarian decision-making along both its dimensions, and that do not rest on the assumption that utilitarian decision-making refers to any unified psychological phenomenon in the everyday context—an assumption that is already put into question by the evidence we have reviewed.

The theoretical framework we propose here makes no such problematic assumptions. Importantly, it avoids classifying the moral judgments of nonphilosophers as ‘utilitarian,’ and does not conceive of proto-utilitarian tendencies in ordinary people in terms of the frequency of such judgments—let alone their frequency in one highly specific moral domain. Instead, our proposed framework understands utilitarian tendencies in terms of the distance of broader patterns of moral dispositions from the paradigmatic concerns of an unqualified utilitarian outlook, leaving open the possibility that one can be more or less utilitarian in some respects yet not in others.

In an important contribution, Conway & Gawronski (2013) used a process dissociation approach to disentangle two distinct factors that can drive prosacrifice judgments: a permissive attitude to directly harming others, and giving greater weight to saving the larger number. This is a significant advance, but it is worth explaining why it does not address the issues we have been raising. Although the two may sound similar, the distinction between a permissive attitude to harm and increased weight to consequences in the context of sacrificial dilemmas does not correspond to our distinction between negative and positive dimensions of utilitarianism. To judge that saving five lives is morally more important than sacrificing the life of another in an emergency situation in no way indicates the kind of impartiality that is at the heart of the positive dimension of utilitarianism, because such an act involves neither self-sacrifice nor regarding distant strangers as having the same moral importance as those dear or near to us. After all, standard sacrificial dilemmas do not typically involve a choice between those close to us and others, and the relevant individuals are all in great proximity. While the link between antisocial traits and greater rates of prosacrifice judgment is likely to be associated with reduced aversion to causing harm rather than increased concern with saving more lives (Conway & Gawronski, 2013), in Kahane et al. (2015) we found that prosacrifice judgments were *not* associated with markers of impartial concern for the greater good even when we controlled for such antisocial traits.^5^

### Existing Individual Difference Scales

An adequate measure of proto-utilitarian psychological tendencies would need to collect responses that are not confined to a specific moral context. This would be hard to achieve effectively using detailed vignettes along the lines of conventional sacrificial dilemmas, since a great many such vignettes would be needed to cover a wide range of scenarios, and this would be unduly burdensome on both participants and researchers. To the extent that we regard proto-utilitarian decision-making tendencies as a fairly stable feature of individuals, a preferable approach would be to administer a scale that could capture individual differences in such tendencies using shorter items. Several such scales purporting to measure utilitarian (or, more broadly, ‘consequentialist’) tendencies have already been developed—for example, the Consequentialist Thinking Scale (Piazza & Sousa, 2014), Baron’s Revised Utilitarianism Scale (U-Scale: Baron, Scott, Fincher, & Emlen Metz, 2015)—which includes parts of the Consequentialist Thinking Scale—and Robinson’s unpublished Consequentialist Scale (Robinson, 2012). However, these scales have broadly the same limitations as the sacrificial dilemmas we discussed above: as with sacrificial dilemmas, they typically only capture the negative dimension of utilitarianism, with characteristic items across the scales including “When we can help some people a lot by harming other people a little, we should do this” (Baron et al., 2015); “Killing someone can be morally right if it is for the greater good” (Baron et al., 2015; Piazza & Sousa, 2014); and “It is never morally justified to cause someone harm” (reverse scored; Robinson, 2012). In other words, these scales largely ignore utilitarianism’s positive impartial ideal and the practical contexts in which it may be manifested (e.g., situations that pit self-interest or concern for those close to us against the well-being of distant strangers). Moreover, formal scale development procedures such as exploratory factor analysis (EFA) and confirmatory factor analysis (CFA) have not been reported for some existing scales (e.g., Baron et al., 2015; Piazza & Sousa, 2014). Using empirical, data-driven, models for scale development is particularly important when trying to measure an abstract concept like utilitarianism because it cannot be assumed a priori that what is conceptually unitary in the philosophical context will also be so in the psychology of ordinary people (Kahane, 2015; see Supplemental Materials for a more extended discussion of existing scales). The need for a new scale is therefore apparent.

## A New Approach to Measuring Proto-Utilitarian Tendencies

Both conceptual considerations and considerable empirical evidence strongly suggest that sacrificial dilemmas are a limited basis for studying utilitarian tendencies, and an adequate scale has not yet been developed. To study utilitarian decision-making in the lay population, a new measure is required. The theoretical framework we outlined above suggests an alternative approach.

First and foremost, an adequate tool for investigating proto-utilitarian tendencies would need to draw on philosophical expertise to ensure that the psychological construct being measured correctly captures the relevant ethical concepts. But it also needs to make sure that the construct maps on to tendencies in general populations rather than the theoretical views of professional philosophers. Consequently, such a tool would need to be a measure of *degrees* of individual differences in utilitarian tendencies instead of an all-or-nothing construct. Few individuals in the lay population are likely to be full-fledged utilitarians, but some may be more utilitarian than others, or may be so only along certain dimensions.

Second, in contrast to both sacrificial dilemmas and existing individual difference questionnaires that purport to measure utilitarian or ‘consequentialist’ tendencies (but which similarly focus on willingness to cause harm), such a measure would need to cover both the ‘negative’ and ‘positive’ aspects of utilitarianism. That is, the scale should also cover the degree to which individuals think about morality and the well-being of others in impartial terms, giving no more (or less) moral priority to the self, as well as to those with whom one has close ties.

The present study aimed to develop a new measure of individual differences in utilitarian tendencies that meets the above desiderata. It also sought to use this new measure to study the relationship between utilitarian tendencies and various other traits to advance our understanding of proto-utilitarian thinking. Because the desiderata include treating utilitarian tendencies as a matter of degree and assessing responses across a range of moral situations (i.e., not only in relation to willingness to violently sacrifice others), we developed a measure more along trait-level individual differences in utilitarian tendencies rather than a measure of an episodic psychological process—that is, a measure of whether or not a subject is engaged in ‘utilitarian decision-making’ at a given point in time. In other words, the measure we developed is akin to measures of individual differences in endorsement of core moral values, such as the Moral Foundations Questionnaire (Graham et al., 2011). Notice, however, that while we aimed to develop a measure of the moral outlook of *individuals*, such a measure would also provide strong (if defeasible) evidence about the moral factors that underlie their moral *judgments* in more specific contexts. For example, if such a measure indicates that someone’s moral outlook is strongly impartial, this would make it more likely that this person’s judgments about, for example, permissible harm, duties of aid, or the stringency of promises, reflect such impartiality.

Given this need to measure responses across a range of moral situations and dimensions, a battery of detailed moral dilemmas would be long and cumbersome. We therefore opted for a short scale measuring responses to a list of brief items. Finally, to ensure that the proposed scale adequately reflects the relevant philosophical concepts and theories, our scale was developed by a joint team of psychologists and moral philosophers. We formulated the pool of items on which the scale is based by conducting a systematic review of the ethical literature; these items were then vetted by leading professional philosophers in the U.S.A. and U.K., both utilitarian and nonutilitarian.

## Scale Development Procedure

### Overview

To develop and validate our scale, we set in advance and then followed a formal scale development procedure to ensure that our measure was both reliable and valid. First, we created an initial pool of items based on the existing literature on the target construct of utilitarianism. After paring the pool down by, for example, eliminating redundancies or unclear items, we submitted the items to an expert panel of academic philosophers for review, and then modified the items in response to their feedback. Third, we recruited a large sample of participants to complete the revised pool of items and then conducted a series of exploratory factor analyses (EFA) to obtain the best factor structure with the best items. Fourth, we conducted a series of confirmatory factor analyses (CFA) to evaluate and refine the best factor structure based on predetermined indices of model fit obtained from recent recommendations in the literature. Fifth, we recruited a new sample of participants to complete the items determined by the CFA and then we confirmed, using this new sample, that the measure had appropriate factor structure and psychometric properties. Sixth, we confirmed that the data fit this model and factor structure better than alternative models (e.g., that a multidimensional model obtained from the CFA accounted for the data better than a one-dimensional model, or vice versa). Seventh, we explored construct validity by testing how scores on the final scale obtained from the previous steps were connected to other established measures. Finally, based on helpful feedback from reviewers of a previous draft of this paper, we investigated external validity by administering the scale to an expert sample of graduate students and academics specializing in moral philosophy. A more extended account of the scale development process can be found in the Supplemental Materials; in the interests of brevity we report only the essential information in the main paper.

### Item Generation

An initial pool of items was generated through a comprehensive survey of the existing literature on utilitarianism. In creating the initial pool of items, several considerations were taken into account. First, we judged it necessary to include items that tapped into the abstract tenets of utilitarianism as well as items that bore on real-world moral judgments that track utilitarian thinking. Second, it was essential to include items that captured both the positive and negative components of utilitarianism: namely, that the right act is the one that impartially maximizes the greater good (positive component), and that this maximization is all there is to morality such that deontological rules and constraints must be rejected when they stand in the way of achieving this goal (negative component). Moreover, we hoped to have a range of items that included: (a) abstract statements of utilitarian belief (b) antiutilitarian views, (c) items reflecting the application of utilitarianism to concrete contexts, and (d) items briefly stating seminal examples or illustrations used by both critics and defenders of utilitarianism. Finally, the items all involved moral judgments of various kinds. For simplicity of presentation, these were phrased using a range of explicit normative terms with synonymous or closely overlapping content such as what is ‘right,’ ‘required,’ or what we ‘should do’ or are ‘obliged to do’ (prior studies suggest that such minor variation in wording has little or no effect on responses; O’Hara, Sinnott-Armstrong, & Sinnott-Armstrong, 2010). When an item could also be interpreted in legal terms, we made it explicit that the question was concerned with what is morally right or wrong rather than legally right or wrong—that is, with what we should do from a moral point of view.

We employed both a ‘bottom-up’ and a ‘top-down’ approach to identifying relevant items. The ‘bottom-up’ approach involved compiling sources from the existing empirical literature purporting to measure utilitarian judgments and extracting relevant citations from their references sections in a systematic fashion (e.g., Greene et al., 2001; Moore, Clark, & Kane, 2008; Piazza & Sousa, 2014; Robinson, 2012). This resulted in more than 200 items, mostly in the form of vignettes or short statements describing moral dilemmas. An initial review of these items revealed considerable redundancy in terms of theoretical content, with the majority of cases clustering around variants of the well-known ‘push’ and ‘switch’ dilemmas (Foot, 1967; Greene, Sommerville, Nystrom, Darley, & Cohen, 2001; Thomson, 1985). We therefore deemed it necessary to perform a ‘top-down’ analysis, as well, to ensure a more robust theoretical foundation. For this analysis, we drew on the philosophical literature as well as the expertise of professional moral philosophers—including members of the present research team—and reviewed both classical and more recent discussions of utilitarianism. Classical statements of the theory included those by Bentham (1789/1983), Mill (1863), and Sidgwick (1901); important recent contributions included work by, for example, Smart and Williams (1973), along with critiques (e.g., Rawls, 1971) and defenses (e.g., Kagan, 1989) of utilitarianism or consequentialism more generally, as well as influential further developments of the theory (e.g., Parfit, 1984). We also took care to include works focusing on the practical implications of utilitarianism (e.g., Singer, 1993). Special emphasis was put on identifying key points of conflict between a strict utilitarian approach and commonsense morality as well as competing ethical theories—conflicts that include willingness to cause harm, break moral rules, compromise virtue or integrity, place limits on the demandingness of morality, and so forth. Finally, after filtering out major redundancies between items, irrelevant items, and poorly worded or confusing items, we were left with a smaller pool of 94 items that were then edited for theoretical clarity and ease of understanding.

### Expert Review

Having generated this pool of 94 items based on initial assessments from within the research team, we then recruited an external panel of leading experts to review the items. Our panel consisted of 11 professional philosophers working in ethics or moral philosophy who had a diversity of viewpoints (including classical act utilitarians, consequentialists who depart from classical utilitarian views, and ethicists who reject consequentialism in all of its forms). Our expert panel included some of the most prominent living contributors to the philosophical literature on utilitarianism, for example, Peter Singer, Shelly Kagan, John Broome, and Alistair Norcross. To ensure that our final set of items would be intelligible to nonphilosophers, we asked our experts to evaluate the items in terms of their brevity, simplicity, and accessibility, while maintaining theoretical specificity. At the same time, we explained that the planned scale was intended to measure utilitarian tendencies in nonphilosophers, such that highly subtle philosophical distinctions that were unlikely to be relevant in such a context should not be emphasized in the assessment of items (see Supplemental Materials for actual text and instructions). In an online survey, each expert was given a list of all 94 statements and asked to indicate “How good do you think this item is for discriminating utilitarian and nonutilitarian views?” (1 = *not at all*; 5 = *very much*),^6^ with space for written comments. Our interest was primarily in the experts’ qualitative feedback and comments on the items, but we also collected the numerical ratings as a complementary source of data. We then removed or modified items to incorporate the experts’ philosophical insights on a case-by-case basis, carefully considering their written comments and their corresponding numerical ratings. We opted for a relatively inclusive pool of items, only dropping items that the experts found particularly unhelpful to discriminating utilitarian and nonutilitarian views. The items that were retained had a mean rating of 3.52 and those that were dropped a mean rating of 2.96. In a substantial number of cases a lower rating reflected ‘fixable’ concerns about the wording of an item rather than intractable concerns about its underlying content; in these cases we opted to revise the items rather than drop them. The specific suggestions of the experts were discussed within the research team until a consensus on exclusion or revised wording was achieved.

These efforts resulted in a smaller pool of 77 items with which to conduct the next stage of the scale development: the exploratory factor analysis in a lay sample. It is worth noting that although the experts were given space to propose new items for inclusion in our scale, none was suggested, indicating that the 94 items provided good coverage of the relevant moral issues. And while the numerical data were not our focus, all of the original items that the experts rated and which were included in the final scale (including modification, if necessary) were rated above the midpoint of the scale, suggesting that they were good items for discriminating utilitarian and nonutilitarian views.

## Study 1

### Ethics Statement

Relevant ethical guidelines were followed and the research was approved through University of Oxford’s Central University Research Ethics Committee, with the reference number MSD-R50145/RE001. Written informed consent was obtained electronically from all participants.

### Participants and Procedure

1009 participants completed the survey online using Amazon Mechanical Turk (MTurk). Participants were excluded from analysis if they completed the survey more than once (in which case only their first attempt was included), or if they failed one or more of five simple attention checks embedded among the items requiring them to “Please click scale point X to confirm you are paying attention.” This left a final sample of 960 participants (489 female, *M*_age_ = 35, *SD* = 12.11), of whom the majority of participants had attended college or higher education (81%). Participants were given the expert-assessed list of 77 items, presented in a semirandomized order. For each item, participants were asked to “indicate how much you agree or disagree with each of the following statements” (1 = *strongly disagree*, 4 = *neither agree nor disagree*, 7 = *strongly agree*).

Our final sample size of 960 was more than adequate. Compared with experimental designs and statistical techniques such as ANOVA where one can compute the required power given the effect size, determination of sample size for factor analysis is notoriously tricky (Mundfrom, Shaw, & Ke, 2005). One approach is to focus on the absolute sample size. While some have suggested a minimum sample size of 250 (Cattell, 1978) or even 100 (Gorsuch, 1983), more recent estimates have suggested that a good sample size is at least 300, and that 1,000 or more is excellent (Comrey & Lee, 1992). Another approach is to focus more on the subject-to-item ratio, or the number of participants for each item used. It is typically accepted that a subject-to-item ratio should be no lower than 5:1 (Gorsuch, 1983), and that 10:1 is appropriate (Everitt, 1975; Nunnally, 1978). Based on these considerations, we therefore decided to recruit 1000 participants. This represented an excellent absolute sample size, and (with 77 items), gave us a good final subject-to-item ratio of 12:1.

### Exploratory Factor Analysis

We first performed a series of exploratory factor analyses (EFA) on the full set of items (77 in total) to ascertain the underlying factor structure. For factor extraction, we used both Kaiser’s criterion (eigenvalues set to 1) in conjunction with inspecting the scree plots. We did this because Kaiser’s criterion tends to overextract factors when the number of variables is large (Linn, 1968). For all EFAs, we used the maximum likelihood estimator with direct oblimin rotation. The first EFA, according to Kaiser’s criterion, yielded 19 factors explaining 57% of the variance in the 77 items whereas the scree plot indicated a 6 factor solution. Inspecting the rotated factor matrix indicated that a number of items did not load onto any factor, as indicated by a factor loading falling under the .30 mark. We excluded these 13 items and reran the EFA. This second EFA indicated that a further 5 items did not load significantly onto any factor. We repeated this process a total of 10 times. The 10th EFA yielded a four-factor solution that explained 43% of the variance in all the items. See Table 1 for the factor solution and for the reliabilities.[Table-anchor tbl1]

### Confirmatory Factor Analysis

Next, using the *lavaan* package in *R*, we performed a Confirmatory Factor Analysis (CFA) on the items obtained from the initial EFA structure (see Table 2 for items). For the CFA, we used the maximum likelihood estimator with robust standard errors and a combination of fit indices to adjudge model fit. There are a plethora of model fit indices at a researcher’s disposal, with disagreement as to what the cut-offs for these indices should be (for an overview, see Hooper, Coughlan, & Mullen, 2008). There are concerns with using only one indicator of model fit (if some indices are more favorable than others, then this could be the only one reported), and so prior to data collection we decided to report four of the most widely acceptable and recommended indices: the chi-square statistic; root mean square error of approximation (RMSEA) and its associated confidence interval; the standardized root-mean-square residual (SRMR), and comparative fit index (CFI; Bentler, 2007; Hu & Bentler, 1999; Kenny, 2015; Kline, 2005). In the process of writing the paper we decided to also report the two parsimony fit indices, AIC and BIC, as recommended by Hooper et al. (2008). Therefore, following the best recommendations in the literature, we conducted and report a combination of six model fit indices: the chi-square statistic; RMSEA and its associated confidence interval; SRMR; CFI; and the parsimony fit indices AIC and BIC (see Table 3). We shall briefly discuss each of these in turn.[Table-anchor tbl2][Table-anchor tbl3]

First, we report the chi-squared test. This is the traditional test used in factor analysis, and indicates the difference between the observed and expected covariance matrices. In the chi-squared test, good model fit is indicated by a nonsignificant result at the level of *p* < .05. Unfortunately, the chi-squared test is very sensitive to sample size, leading to problems of false-negatives in small sample sizes and false-positives in large sample sizes. Given this, it is necessary to supplement the chi-squared test with the better indices below, and while we report the chi-square test it is these other (better) indices on which we focused when evaluating model fit.

Second, we use root mean square error of approximation (RMSEA). This an absolute measure of fit that adjusts for sample size when chi-squared tests are used. The RMSEA yields values ranging from 0 to 1, with smaller values indicating better fit. While traditionally it has been suggested that an RMSEA of ≤.08 indicates good fit, this has been revised to suggest that values closer to ≤.06 are better (Hu & Bentler, 1999), and should not exceed .07 (Steiger, 2007). One of the key benefits of using RMSEA is that a confidence interval is available for its value, and recommendations suggest that this should be reported in addition, and that in a well-fitting model the lower the values the better, with the upper limit being ≤.08 (Hooper et al., 2008; Hu & Bentler, 1999; Kenny, 2015; Steiger, 2007).

Third, we report the standardized root-mean-square residual (SRMR), which is again an absolute measure of fit and represents the square root of the discrepancy between the observed covariance matrix and the hypothesized covariance matrix. Values range from 0 to 1, with smaller values indicating better fit and a value of ≤.08 indicating acceptable fit (Hooper et al., 2008; Hu & Bentler, 1999; Kenny, 2015; Steiger, 2007).

Fourth, we used the comparative fit index (CFI): a relative fit index that examines the discrepancy between the actual data and the hypothesized model, making adjustment for the issues of sample size that can be problematic in the chi-squared test of model fit. CFI is one of the most popularly reported fit indices and is least affected by sample size (Fan, Thompson, & Wang, 1999). Like RMSEA and SRMR, CFI yields values from 0 to 1, but unlike the others, for the CFI it is higher values that indicate better fit. Traditionally, CFI scores of ≥.90 have been taken to indicate acceptable fit, but this has since been revised to suggest that this is too lenient and that for good fit, scores should be ≥.95 (Hu & Bentler, 1999) It is this more conservative level we settled upon for our analyses.

Fifth and sixth, when comparing the eventual models we looked at the parsimony fit indices of the Akaike Information Criterion (AIC: Akaike, 1974) and the closely related Bayesian Information Criterion (BIC: Schwarz, 1978). These indices indicate which model is more parsimonious, with smaller values indicating good fit. In contrast to the other indices, there is no cut-off here, but the recommendation is that when comparing two models, that which produces the lowest AIC and BIC score is the better fitting model (Hooper et al., 2008; Kenny, 2015).

Note that all of these are recommendations rather than determinative rules; an overall decision should be made through considering the results across the different indices. Nevertheless, we provisionally agreed upon the following cut-off criteria as indicative of adequate model fit: RMSEA ≤ .07, SRMR ≤ .08, and CFI ≥ .95 (Bentler, 2007; Hooper et al., 2008; Hu & Bentler, 1999; Kenny, 2015). In addition, we decided to look at the AIC and BIC scores because even though there are no cut-off criteria, when comparing two models one can see which has the lowest AIC/BIC scores, thus indicating a better fit. By using such a range of recommended fit indices, we aimed to improve confidence in our results. We first performed the CFAs for the four above factors individually, and then, once all items were finalized, we entered the factors into a CFA simultaneously. We had two main aims in our CFA analyses: to find a factor structure that would give the best statistical fit, but also to produce a short scale.

### Individual Factor CFAs

#### Factor 1 (‘Impartial Beneficence’)

Factor 1 contained 11 items that converged around the concept of impartial beneficence, consisting of items such as “It is morally wrong to keep money that one doesn’t really need if one can donate it to causes that provide effective help to those who will benefit a great deal.” The CFA for Factor 1 with 11 items returned fit statistics that were less than adequate and did not meet our cut-off criteria, χ^2^(44) = 401.518, *p* < .001, CFI = .855, RMSEA = .092 [.08, .10], SRMR = .059, AIC = 38445.33, BIC = 38552.41. To increase factor stability, we decided to exclude all items with factor loadings lower than .40. As a result, item 63 (standardized factor loading = .39) and 48 (standardized factor loading = .39) were dropped. Dropping these two items resulted in a 9-item solution that did fit better but which still did not meet our cut-off criteria, χ^2^(27) = 258.331, *p* < .001, CFI = .889, RMSEA = .094 [.084, .105], SRMR = .054, AIC = 31346.90, BIC = 31434.51. Looking over the model modification indices, there was evidence of correlated error variances between items 77 and 73. Both items seemed to measure rejection of the Doctrine of Double Effect (i.e., that it can be permissible to cause harm as a side effect but not as an intended outcome), but because item 77 was much longer and potentially confusing, we decided to remove item 77. The resultant modification indices showed better fit for the 8-item solution, but again still did not meet our cut-off criteria, χ^2^(20) = 163.687, *p* < .001, CFI = .919, RMSEA = .087 [.075, .099], SRMR = .046, AIC = 27877.68, BIC = 27955.55. We next removed items 14 and 15 on theoretical grounds: item 14 was similar to, but less precise than, item 16; and item 15 was not theoretically critical because questions about self-defense are not central to debates about utilitarianism. After deleting these two items, the resultant 6-item model showed better model fit but still did not meet our cutoff criteria, χ^2^(9) = 83.69, *p* < .001, CFI = .926, RMSEA = .093 [.075, .112], SRMR = .047, AIC = 21284.17, BIC = 21342.57. Items 61 and 62 both concerned impartiality in helping those close to us, but because item 61 had the weakest loading and item 62 was better as an abstract statement of impartiality, we next deleted item 61, which led to a 5-item model with substantially improved fit, χ^2^(5) = 26.83, *p* < .001, CFI = .974, RMSEA = .067 [.044, .093], SRMR = .032, AIC = 17845.76, BIC = 17894.43. This 5-item solution had the lowest AIC and BIC of the models tested, and met our cut-offs for the CFI (≥.95), RMSEA (≤.07), and SRMR (≤.08). Therefore, items 16, 17, 11, 73, and 62 were included in the final CFA for the first construct. Because these items seemed to tap into the aspect of utilitarianism that seeks to impartially maximize welfare and the greater good, even at expense to oneself, we labeled this factor *Impartial Beneficence*.

#### Factor 2 (‘Instrumental Harm’)

Next, we conducted a CFA on Factor 2. Factor 2 consisted of 9 items that seemed to tap the construct of willingness to endorse instrumental harm, including items such as “It is morally right to harm an innocent person if harming them is a necessary means to helping several other innocent people.” The model with all 9 items showed acceptable model fit, but did not meet all of our cut-off criteria, χ^2^(27) = 149.01, *p* < .001, CFI = .940, RMSEA = .069 [.058, .080], SRMR = .045, AIC = 30732.32, BIC = 30819.92. We wanted to improve the model fit and reduce the number of items to be more comparable to the first factor, and so because item 70 had fairly low loadings, we removed it from the next CFA to have an 8-item solution, χ^2^(20) = 131.43, *p* < .001, CFI = .94, RMSEA = .076 [.064, .089], SRMR = .048, AIC = 27289.42, BIC = 27367.29. This model still did not meet our cut-off criteria. Next, we noted that the error variance for item 59 was correlated with the error variances of three other items (67, 20, and 52), indicating that there was another factor that was not accounted for by our model which explained a significant amount of variance between these three items. To gain as clean a factor structure as possible, we next removed item 59 from the CFA. This 7-item solution resulted in decent model fit, χ^2^(14) = 70.25, *p* < .001, CFI = .97, RMSEA = .065 [.050, .080], SRMR = .037, AIC = 23803.09, BIC = 23871.23. However, items 43 and 20 still shared a significant amount of error variance and so to obtain as clean a factor structure as possible, we deleted item 43, which was theoretically less fundamental because it referred to political restriction of freedom rather than to causing acute harm. The resultant six-item CFA showed good model fit, χ^2^(9) = 50.149, *p* < .001, CFI = .976, RMSEA = .07 [.051, .088], SRMR = .03, AIC = 20362.66, BIC = 20421.06. We remained concerned, however, that two items (68 and 69) in the model were too similar in both content and language and so we next excluded the weaker loading item (item 69). This five item factor-solution showed good model fit: χ^2^(5) = 26.870, *p* < .001, CFI = .978, RMSEA = .068 [.044, .094], SRMR = .031, AIC = 17416.26, BIC = 17464.93. Finally, we removed item 52—the weakest loading—because it included elements relating both to instrumental harm and to impartiality and was therefore somewhat theoretically removed from the other items. Deleting this item resulted in a four-item solution with excellent fit, meeting all of the recommended fit index cut-off levels we specified before data collection (CFI ≥ .95; RMSEA ≤ .07; SRMR ≤ .08), and having the lowest AIC and BIC of the different solutions tested: χ^2^(2) = 4.47, *p* = .11, CFI = .997, RMSEA = .036 [.00, .08], SRMR = .014, AIC = 13766.89, BIC = 13805.82. Therefore, this four-item solution with the items 20, 67, 68, and 72 was chosen as the final solution for the second factor. Because the second factor tapped support of allowing harm in the service of the greater good, we labeled it *Instrumental Harm*.

#### Factor 3 (‘Anti-Traditional Morality’)

Factor 3 consisted of 10 items that were concerned with traditional morality—a set of deontological ideas associated with conservative thought, such as retributive punishment, sexual morality, human dignity, and an absolutist view of social and moral rules. An example of an item from this factor is “Criminals should receive the punishment they deserve—even if this will not protect the public or deter crime in the future.” The CFA for the third factor with 10 items showed poor model fit, χ^2^(35) = 417.182, *p* < .001, CFI = .830, RMSEA = .107 [.098, .116], SRMR = .067, AIC = 34950.38, BIC = 35047.72. Looking at the factor loadings, items 21 and 23 had very low factor loadings (both = .25), and so we removed them from the model. Removing these two items gave a 8-item model with slightly better fit, though it was still substandard, χ^2^(20) = 252.735, *p* < .001, CFI = .884, RMSEA = .110 [.098, .122], SRMR = .058, AIC = 28576.34, BIC = 28654.21. The modification indices highlighted a strong correlation between the error variances for items 27 and 26, and so we decided to remove the item with more error variance (item 26). Dropping this item gave us a 7-item model that had better fit but still did not meet our cut-off criteria, χ^2^(14) = 92.073, *p* < .001, CFI = .938, RMSEA = .076 [.062, .091], SRMR = .041, AIC = 25144.06, BIC = 25212.20. To improve fit, we next deleted item 28, which in addition to being the lowest-loading item, also was more theoretically distinct by focusing more on political authority than morality. The resulting six-item model fit the data well, χ^2^(9) = 49.617, *p* < .001, CFI = .962, RMSEA = .069 [.051, .088], SRMR = .034, AIC = 21653.56, BIC = 21711.97. This model consisting of items 2, 4, 18, 25, 27, and 57 met all of the recommended fit index cut-off levels we specified before data collection (CFI ≥ .95; RMSEA ≤ .07; SRMR ≤ .08), and had the lowest AIC and BIC of the different solutions tested. Therefore, this was chosen as the final solution for the third factor, and because this factor tapped the rejection of traditional deontological moralities we labeled it *Anti-Traditional Morality*.

#### Factor 4 (‘Truth-Telling and Promise-Keeping’)

Finally, we looked at Factor 4, which consisted of 5 items concerning truth-telling and promise keeping, such as “It is morally wrong to break promises even if this would bring about good outcomes” and “It is morally permissible to lie if doing so would help others a great deal.” In addition to explaining the least variance, Factor 4 also represented a group of moral views that are not distinctive of utilitarianism given that few hold that it is never permitted to lie or break promises, limiting its usefulness to the scale. Nonetheless, for completeness we again conducted a CFA. The first CFA with all 5 items showed weak model fit, χ^2^(5) = 181.942, *p* < .001, CFI = .882, RMSEA = .192 [.169, .216], SRMR = .070, AIC = 15988.92, BIC = 16037.59. Because item 46 was the weakest loading and shared a lot of variance with items 45 and 47, we next excluded this item. This four-item solution was better but still had unacceptable model fit, χ^2^(2) = 30.773, *p* < .001, CFI = .975, RMSEA = .122 [.087, .162], SRMR = .030, AIC = 12743.30, BIC = 12782.24. Given both prior theoretical concerns (that issues relating to honesty and promise-keeping are not central, or even particularly important, to utilitarianism) and the suboptimal results from the CFA, we therefore decided to not include this factor in the final scale.

### Overall Factor Solutions

We first tested a three-factor solution by conducting a CFA with all three retained factors and their corresponding manifest items entered into the model simultaneously (Factor 1: items 11,16,17,73,62; Factor 2: items 20,67,68,72; Factor 3: items 2,4,18,25,27,57) The resulting model fit was acceptable, but still did not meet our cut-off criteria, χ^2^(87) = 352.167, *p* < .001, CFI = .913, RMSEA = .056 [.050, .063], SRMR = .056, AIC = 53253.596, BIC = 53414.205. Given this, we compared this factor model to one where all 15 items loaded onto a single factor. This model produced a significantly worse model fit, χ^2^(90) = 2256.238, *p* < .001, CFI = .287, RMSEA = .158 [.153, .164], SRMR = .159, AIC = 55151.666, BIC = 55297.674. The one-factor model provided extremely poor fit, empirically supporting our theoretical basis for believing that a multifactor solution would best characterize utilitarian tendencies in the lay population. At the same time, these results suggested that a three-factor might not be the most appropriate multifactor solution.

Given that the three-factor solution did not meet the recommended threshold (specifically for the CFI), we considered whether a two-factor solution consisting of Impartial Beneficence and Instrumental Harm might be more appropriate. This two-factor model demonstrated excellent fit: χ^2^(26) = 84.914, *p* < .001, CFI = .967, RMSEA = .049 [.037, .060], SRMR = .040, AIC = 31610.403, BIC = 31702.875 (see Table 4 for Factor Loadings and Table 5 for Final Items). Recall that we had followed recommended cut-off criteria of CFI ≥ .95, RMSEA ≤ .07, SRMR ≤ .08, and the use of AIC and BIC to see which model had the lowest value, thus indicating better fit (Bentler, 2007; Hooper et al., 2008; Hu & Bentler, 1999; Kenny, 2015). Unlike the three-factor model, the two-factor model met the CFI criterion. Moreover, compared with a three-factor model, the two-factor model consisting of Impartial Beneficence and Instrumental Harm had superior fit indices across the board: it had a higher CFI (.967 vs. 913); a lower RMSEA (.049 vs. .056); a lower SRMR (.040 vs. .056); a lower AIC (31610.403 vs. 53253.596); and a lower BIC (31702.875 vs. 53414.205).[Table-anchor tbl4][Table-anchor tbl5]

A two-factor model with only Impartial Beneficence and Instrumental Harm was clearly better than a three-factor model, but might an alternative two-factor model be even better? We compared our two-factor model consisting of Impartial Beneficence and Instrumental Harm with two-factor models consisting of Impartial Beneficence and Anti-Traditional Morality, and instrumental harm and Anti-Traditional Morality. Results indicated that our selected model consisting of Impartial Beneficence and Instrumental Harm showed better fit than the other versions. A two-factor model consisting of Impartial Beneficence and Anti-Traditional Morality did not meet our cut-off criteria for the CFI, and indeed had worse fit indices across the board: χ^2^(43) = 159.365, *p* < .001, CFI = .941, RMSEA = .053 [.044, .062], SRMR = .045, AIC = 39493.458, BIC = 39605.397. The same was observed for a two-factor model consisting of Instrumental Harm and Anti-Traditional Morality: χ^2^(34) = 199.499, *p* < .001, CFI = .22, RMSEA = .071 [.062, .081], SRMR = .062, AIC = 35417.079, BIC = 35519.285.

In summary, then, the two-factor model consisting of only Impartial Beneficence and Instrumental Harm (and not the Anti-Traditional Morality factor) showed substantially better fit across the different indices than a unidimensional model, a three-factor model with all three factors, a two-factor model consisting of only Impartial Beneficence and Anti-Traditional Morality, and a two-factor model consisting of only Instrumental Harm and Anti-Traditional Morality. In fact, the two-factor Impartial Beneficence and Instrumental Harm model was the only one to meet all the recommended fit indices we had settled on at the start of the project based on the recent directives in the field (Bentler, 2007; Hooper et al., 2008; Hu & Bentler, 1999; Kenny, 2015).

There is therefore a strong psychometric basis for choosing a two-factor model. Such a decision, however, can also be supported on theoretical grounds. The Anti-Traditional Morality factor largely taps the rejection of deontological absolutism and traditional moral rules (e.g., those relating to punishment or sexual purity). Although such views are very distant from utilitarianism, this is not in and of itself diagnostic for distinguishing utilitarians from nonutilitarians. While utilitarians may have been historical pioneers in advocating for sexual freedom and the rejection of rigid moral norms, such moral views are today widely shared within secular morality, including by those holding resolutely nonutilitarian views such as libertarianism or pluralist liberal moralities focusing on rights. Contemporary defenders of Kantian ethics also often reject an absolutist interpretation of the approach, and dismiss Kant’s rigid views about, for example, sexuality and suicide. A high score on the Anti-Traditional Morality factor therefore indicates acceptance of a broadly secular morality but does not yet amount to an interesting move in a distinctively utilitarian direction. Indeed, the comparatively poor fit of the three-factor solution may suggest that it is unhelpful to treat moral absolutism as the key contrast with utilitarianism, as some previous scales do.

## Study 2: Scale Validation

In Study 1 we established a provisional 2-factor *Oxford Utilitarianism Scale* (OUS). In Study 2 we sought to confirm—and if needed, refine—this scale. To confirm the factor structure and establish contrast validity, we recruited a new set of participants to complete the scale and a number of theoretically related constructs. This allowed us to perform a second CFA using the new dataset, and to examine how well scores on the OUS related to existing measures of utilitarianism.

### Participants and Procedure

Three hundred participants were recruited online using MTurk. Eighteen participants were excluded from analysis for answering a simple attention check incorrectly or not completing the survey, leaving a final sample of 282 participants (178 female, *M*_age_ = 39, *SD* = 12.66). The majority of participants had attended college or higher education (80%). As before, participants rated how much they agreed or disagreed with the statements (1 = *strongly disagree*, 7 = *strongly agree*). Participants completed the provisional OUS first (with the Anti-Traditional Morality items, to confirm, with a new sample, the appropriateness of excluding those items as had been decided before) and then moved on to complete a series of other measures. These measures were designed both to assess construct validity and to show how the OUS can shed light on previously found relationships between individual differences and (so-called) utilitarianism. To prevent order effects, these other measures were presented in a randomized order, with demographic questions and questions on political and religious belief at the end.

### Confirmatory Factor Analysis

Beginning by looking at the factors separately, the first factor of Impartial Beneficence showed excellent model fit, χ^2^(5) = 3.754, *p* = .59, CFI = .1.00, RMSEA = .000 [.000, .071], SRMR = .021, AIC = 5281.130, BIC = 5317.549. Similarly, the second factor of Instrumental Harm showed excellent fit, χ^2^(2) = 3.563, *p* = .168, CFI = .994, RMSEA = .053 [.00, .140], SRMR = .022, AIC = 4027.463, BIC = 4056.599. Finally, the third factor of Anti-Traditional Morality showed weaker but acceptable fit, meeting the CFI and SRMR criteria, but not the RMSEA: χ^2^(9) = 24.549, *p* = .004, CFI = .969, RMSEA = .078 [.042, .116], SRMR = .038, AIC = 6106.079, BIC = 6149.782.

Next, we looked at the overall factor solutions. The two-factor CFA model selected from Study 1 with Impartial Beneficence and Instrumental Harm showed excellent fit: χ^2^(26) = 39.550, *p* = .043, CFI = .975, RMSEA = .043 [.008, .069], SRMR = .048, AIC = 9302.380, BIC = 9371.576. As in Study 1, our selected model performed substantially better than all the other alternatives, having superior fit across all indices and being the only one to meet all of the recommended cut-offs. Our selected model with Impartial Beneficence and Instrumental Harm performed better than a one-factor model, χ^2^(90) = 704.826, *p* < .001, CFI = .454, RMSEA = .156 [.145, .166], SRMR = .154, AIC = 15923.795, BIC = 16033.052; better than a three-factor model, χ^2^(87) = 184.047, *p* < .001, CFI = .914, RMSEA = .063 [.050, .076], SRMR = .070, AIC = 15409.015, BIC = 15529.198; better than a two-factor model with only Impartial Beneficence and Anti-Traditional Morality, χ^2^(43) = 89.527, *p* < .001, CFI = .942, RMSEA = .062 [.044, .080], SRMR = .061, AIC = 11387.434, BIC = 11471.198; and better than a two-factor model with only Instrumental Harm and Anti-Traditional Morality, χ^2^(34) = 91.792, *p* < .001, CFI = .929, RMSEA = .078 [.059, .097], SRMR = .070, AIC = 10133.293, BIC = 10209.773. As in Study 1, a two-factor model with Impartial Beneficence and Instrumental Harm had better fit than all the other alternatives, and indeed was the only one to meet all the recommended fit criteria.

### The Oxford Utilitarianism Scale (OUS)

Having assessed the scale on both theoretical and empirical grounds, we therefore arrive at a final scale consisting of 9 items in two subscales. The first subscale—*Impartial Beneficence* (OUS-IB)—consists of 5 items that all tap endorsement of the impartial maximization of the greater good, even at the cost of personal self-sacrifice:

1 If the only way to save another person’s life during an emergency is to sacrifice one’s own leg, then one is morally required to make this sacrifice.
2 From a moral point of view, we should feel obliged to give one of our kidneys to a person with kidney failure since we do not need two kidneys to survive, but really only one to be healthy.
3 From a moral perspective, people should care about the well-being of all human beings on the planet equally; they should not favor the well-being of people who are especially close to them either physically or emotionally.
4 It is just as wrong to fail to help someone as it is to actively harm them yourself.
5 It is morally wrong to keep money that one doesn’t really need if one can donate it to causes that provide effective help to those who will benefit a great deal.

The second subscale was labeled *Instrumental Harm* (OUS-IH). This subscale consists of 4 items that all tap a willingness to cause harm to bring about the greater good:

1 It is morally right to harm an innocent person if harming them is a necessary means to helping several other innocent people.
2 If the only way to ensure the overall well-being and happiness of the people is through the use of political oppression for a short, limited period, then political oppression should be used.
3 It is permissible to torture an innocent person if this would be necessary to provide information to prevent a bomb going off that would kill hundreds of people.
4 Sometimes it is morally necessary for innocent people to die as collateral damage—if more people are saved overall.

### Construct Validity

For the final stage of our scale development procedure, we used the same dataset as was used for the second CFA (*N* = 282) to assess the construct validity of the OUS. Since ‘proto-utilitarian tendencies’ is a new construct and existing measures are flawed, there is no straightforward way to validate the scale. We decided to assess construct validity with three key tests assessing convergent validity (i.e., whether things that are theoretically connected are in fact connected in the data). Specifically, if our OUS scale is measuring what it should be measuring, (a) overall OUS scores should be associated with the extent of agreement with an explicit statement of a utilitarian approach to ethics, (b) OUS-IH scores should be associated with prosacrifice responses in sacrificial moral dilemmas, and (c) OUS-IB scores should be associated with responses in ‘greater good’ moral dilemmas that capture participants’ endorsement of self-sacrifice and impartiality in morality (see Table 6 for all Correlations). Note that our focus here is primarily on convergent rather than divergent validity. We have of course developed an overall OUS scale and because scores on the two subscales are positively but weakly correlated, it would make sense if—in addition to the primary relationship between the OUS-IB and the ‘greater good’ dilemmas—there is also a weak positive relationship between the OUS-IH and these dilemmas. What is critical for our 2D model is not that there is absolutely no relationship between instrumental harm and impartial beneficence, but that they are independent and dissociable factors.[Table-anchor tbl6]

We have focused our assessment of construct validity by comparing the OUS with other measures that have been claimed, however accurately or inaccurately, to directly measure utilitarianism. That said, it is prudent to note that the distinction we draw below between measures directly assessing construct validity and those showing how the OUS works in practice could be seen as somewhat arbitrary, given that nearly all previous psychological work on utilitarianism has ignored impartial beneficence. Both sets of measures are important and meaningful in confirming the scale’s validity, but for the specific purpose of assessing construct validity, we begin by looking at purported measures of utilitarianism rather than associated constructs.

### Explicit Utilitarianism

Our first key test of construct validity was to ensure that scores on the OUS were associated with agreement of an explicitly utilitarian moral philosophy. Participants were asked to indicate how much they agreed or disagreed with the following statement:

The only thing that determines whether an act is morally right is whether, out of the available options, it is the act that would lead to the most happiness and the least suffering in the world, taking into account the welfare of all sentient beings, whether human or animal. An act that doesn’t maximize welfare in this way is morally wrong. On this moral view, no one counts for more than anyone else: our own interests and needs, and the interests and needs of our family and friends, never count for more than the interests and needs of any other person, however distant from us. Finally, on this view the only thing that matters is how our actions affect the amount of happiness in the world. It is always morally right to break a rule or principle if doing so would lead to the better outcome.

Note that this explicit description of utilitarianism is well-grounded theoretically and the one that we took as our standard when developing this scale and thus, to the extent that scores on the OUS reflect endorsement of specifically utilitarian moral thinking, they should correlate positively with agreement with this statement of explicit utilitarianism. This was the case. Agreement with the statement of explicit utilitarianism was associated with higher scores on the OUS overall, *r* = .35, *p* < .001, and for each of the two subscales: the Impartial Beneficence subscale, *r* = .37, *p* < .001 and, more weakly, the Instrumental Harm subscale, *r* = .16, *p* < .001. A test of significance between these two correlations (Steiger, 1980) showed that these correlations were significantly different (*Z* = 2.84, *p* = .005), providing empirical support for our theoretically grounded claim that it is impartial beneficence rather than instrumental harm that is at the core of a distinctively utilitarian outlook.^7^

### Sacrificial Moral Dilemmas

As just described, our first key test was to look at how overall OUS scores were associated with a statement of explicit utilitarianism. Our next two tests were focused more on providing convergent validity for the two subscales in isolation, starting with responses to ‘trolley-style’ sacrificial moral dilemmas. Although there are serious concerns about identifying utilitarian judgments with prosacrifice judgments in such dilemmas (Kahane, 2015), such sacrificial dilemmas do seem to track the endorsement of instrumental harm. To the extent that our Instrumental Harm subscale directly measures this negative component of utilitarianism—a willingness to cause harm for the greater good—there should be a significant, positive association between scores on the overall OUS (likely driven by the OUS-IH) and endorsement of the sacrificial action in these trolley-style sacrificial dilemmas.

To assess the convergent validity of the OUS through the association with sacrificial dilemmas, we used three dilemmas involving ‘up-close-and personal’ harm that were adapted from previous research (Moore et al., 2008). The dilemmas we used included, and were inspired by, the classic Footbridge case, in which one can save five people from a runaway trolley only by pushing another person onto the tracks, leading to that person’s death (see Supplemental Materials for a full description). For each of the three dilemmas, participants were asked “How wrong would it be to [perform the ‘utilitarian’ act, for example, push the stranger in the Footbridge case]?” (1 = *not at all wrong*, 7 = *extremely wrong*). These ratings across the three dilemmas were combined into a single reliable measure of participants’ ‘utilitarianism’ (viz., endorsement of instrumental harm) in sacrificial dilemmas (α = .74), where lower scores indicated higher ‘utilitarianism.’ Confirming the validity of the OUS, participants who scored higher on the OUS-IH subscale, *r* = −.32, *p* < .001 and the OUS overall, *r* = −.34, *p* < .001 were more likely to endorse the sacrifice in these dilemmas.

Recall that our focus here was on the convergent validity of the OUS-IH subscale with responses in sacrificial dilemmas, because the OUS-IH is supposed to measure instrumental harm and this is also measured in sacrificial dilemmas. It was less important, but nonetheless interesting, to determine if scores on the OUS-IB were associated (positively or negatively) with responses to these dilemmas. We approached this question in two ways. First, we looked at the correlations between the sacrificial dilemmas and the OUS-IB and found that participants who scored higher on the OUS-IB were also—perhaps surprisingly—more likely to endorse the sacrificial option in the dilemmas, *r* = −.21, *p* < .001. The strengths of correlations between the dilemmas and the two subscales were not significantly different (*Z* = −1.48, *p* = .14). Second, we looked at wrongness ratings for the sacrificial action in the dilemmas by categorizing participants based on whether they were high (i.e., above the scale midpoint) or low (i.e., below the scale midpoint) on both, either, or neither, of the two OUS subscales. To do this we coded participants based on whether they were above or below the midpoint on each of the scales separately, and then entered this into a 2 (OUS-IB: Low vs. High) × 2 (OUS-IH: Low vs. High) between-subjects ANOVA. For convenience we shall call participants who scored high on both subscales “utilitarians” (*N* = 50), and those who scored low on both subscales “deontologists” (*N* = 118). Those who scored high on Impartial Beneficence but low on Instrumental Harm are referred as “impartials” (*N* = 67), and those who scored high on Instrumental Harm but low on Impartial Beneficence as “instrumentalists” (*N* = 47). Note that these labels are simply intended to make the following results more easily interpretable for the reader, and should not be taken as firm categorizations. We observed a main effect on wrongness by whether participants were above or below the midpoint on the OUS-IH, *F*(1, 278) = 23.64, *p* < .001, η^2^ = .08, and a (weaker) main effect based on the OUS-IB, *F*(1, 278) = 4.30, *p* = .04, η^2^ = .02, but no interaction between OUS-IB and OUS-IH, *F*(1, 278) = 0.17, *p* = .68, η^2^ = .00. “Utilitarians” (i.e., those high on both IB and IH) thought the sacrifice to be the least wrong of all four groups, regarding it as significantly less wrong than “impartialists” (i.e., those high only on IB but low on IH; *p* = .01), and “deontologists” (those low on both IB and IH; *p* < .001). There was no difference between “utilitarians” and “instrumentalists” (*p* = .73). Deontologists (low on both IB and IH) thought the sacrifice to be most wrong, significantly more so than utilitarians (*p* < .001), impartialists (*p* = .04), and instrumentalists (*p* < .001). Overall, then, both Impartial Beneficence and Instrumental Harm were associated with thinking the sacrificial action to be less wrong, but unsurprisingly this was stronger for Instrumental harm. While there was no significant interaction between the two dimensions, it is noteworthy that utilitarians high on both dimensions thought the sacrifice to be the least wrong, and deontologists low on both dimensions thought it to be the most wrong.

The significant association we observed between prosacrifice judgments and the OUS-IB (even if weaker than the association with the OUS-IH) is somewhat puzzling given that that in some of our previous work (Kahane et al., 2015) we found no relation between such judgments and a battery of ‘greater good’ dilemmas, a vignette-based measure of the ‘positive’ dimension of utilitarianism (see below). One possibility is that the first-person presentation of the dilemmas in the previous study (e.g., ‘should you push the man?’) had something to do with the observed difference, since for consistency all dilemmas here were presented in the third-person form (e.g., ‘should Adam push the man?’). Further research is required to assess such possibilities.

### ‘Greater Good’ Moral Dilemmas

Our final key test of construct validity was to establish the convergence between OUS-IB and overall OUS scores with responses to dilemmas tapping participants’ endorsement of self-sacrifice and impartiality in morality. To accomplish this, we used three ‘greater good’ dilemmas designed by Kahane et al. (2015) to directly pit an explicit utilitarian action promoting the greater good against a narrower, more partial moral view that allows us to give priority to self, family, and country. For each item participants were asked to rate how wrong it would be for someone to perform the *non*-utilitarian action (1 = *not at all wrong*; 7 = *very wrong*), such that higher scores indicated greater utilitarianism (in contrast to the sacrificial dilemmas). Confirming our construct validity, endorsement of the utilitarian action in these greater good dilemmas was significantly associated with scores on the OUS-IB subscale, *r* = .50, *p* < .001 and with OUS scores overall, *r* = .40, *p* < 001.

We also looked at the correlation between responses to the greater good dilemmas and the other subscale, OUS-IH. Interestingly, there was no relationship between scores on the OUS-IH, *r* = .07, *p* = .26 and responses to the greater good dilemmas, and the correlations between the OUS-IB and OUS-IH with the dilemmas were significantly different (*Z* = 6.04, *p* < .001). Similar results were observed when looking at perceived wrongness of the nonutilitarian act in the greater good dilemmas based on whether participants were high (i.e., above the scale midpoint) or low (i.e., below the scale midpoint) on both, either, or neither, of the two subscales. We observed a main effect on wrongness by whether participants were above or below the midpoint on the OUS-IB, *F*(1, 278) = 34.78, *p* < .001, η^2^ = .11, but no significant effect of the OUS-IH, *F*(1, 278) = 3.16, *p* = .08, η^2^ = .01, and no interaction between OUS-IB and OUS-IH, *F*(1, 278) = 0.43, *p* = .51, η^2^ = .00. “Utilitarians” (high on both IB and IH) thought that failing to perform the utilitarian action was the most wrong of all four groups, thinking it significantly more wrong than “instrumentalists” (high on IH but low on IB; *p* < .001), and “deontologists” (low on both IB and IH; *p* < .001). Similarly, “impartialists” (high on IB but low on IH) thought that failing to perform the utilitarian action was more wrong than instrumentalists (*p* = .006) and deontologists (*p* < .001); but there was no difference between utilitarians and impartialists (*p* = .10), nor between impartialists and deontologists (*p* = .41). Higher levels of impartial beneficence were associated with more characteristically utilitarian responses in the greater good dilemmas, but this was independent of endorsement of instrumental harm. Overall, then, results from both correlational analyses and an ANOVA revealed that while sacrificial judgments were correlated with scores on the OUS-IB, more concrete cases of impartial beneficence were not associated with instrumental harm. These results again highlight that while there is a connection between the utilitarian components of instrumental harm and impartial beneficence, they appear to have relatively distinct psychological correlates.

Finally, it is important to note that of the 5 items in the OUS-IB subscale, 3 are concerned with self-sacrifice for the greater good (1, 2, 5), one with impartiality with respect to strangers versus those close to you (3), and one with the act/omission distinction (4). The three greater good dilemmas we used here covered self-sacrifice to aid distant strangers, self-sacrifice to prevent harm to animals, and refusal to give priority to one’s own country in the context of charitable donation. The OUS-IB subscale was significantly correlated with all 3 dilemmas (*r*s > .31, *p*s < .001), suggesting that it measures both the element of self-sacrifice and the rejection of special duties to those closely associated with you.

## Study 3: Expert Validation

In Study 2, we established construct validity by showing that both the OUS overall and its two subscales were strongly correlated with the degree to which participants endorsed an explicit statement of utilitarianism and that each of the subscales was associated in the appropriate direction with more specific measures of the positive and negative dimensions of utilitarianism (e.g., the OUS-IH with prosacrifice responses to sacrificial dilemmas and the OUS-IB with more impartial responses in the greater good dilemmas).

To provide further validation of the construct, we compared OUS scores with the self-reported moral views of experts in moral philosophy. To the extent that the OUS is a reliable measure of utilitarian tendencies, it should strongly correlate with the degree to which such experts self-identify as utilitarian. Notice, however, that the OUS was designed as a measure of the moral outlook of ordinary people, not as a tool enabling fine-grained classification of the ethical views of moral philosophers.

Collecting responses from experts also allowed us to investigate another question. While both the OUS-IB and OUS-IH were strongly correlated with explicit endorsement of utilitarianism (*r* = .81 and *r* = .70, respectively), they were only weakly associated with each other (*r* = .14), supporting our hypothesis that these two dimensions of utilitarianism are nevertheless independent factors in the psychology of ordinary people. This independence might raise questions about the rationale for including both dimensions in a single scale. However, the conceptual unity of the scale would be demonstrated if the two dimensions were closely linked in expert moral philosophers who either explicitly endorse or reject utilitarianism. We predicted that the two subscales *would* be strongly correlated in a sample of expert moral philosophers.

### Method

#### Participants

Our sample of experts in moral philosophy were recruited through an email sent to the following mail-lists associated with ethics and moral philosophy: the Future of Humanity Institute, the Centre for Effective Altruism, the Ethox Centre, and the Uehiro Centre for Practical Ethics at the University of Oxford; the Anscombe Bioethics Centre for Medical Ethics; the Bioethics Centre at Monash University; The Hastings Center (bioethics research institute); the Yeoh Tiong Lay Centre for Politics, Philosophy & Law at King’s College London; and BioEdge.org. These groups were purposely selected to consist of people who work extensively on questions of ethics and moral philosophy, and care was taken to include both utilitarian-leaning groups (e.g., the Future of Humanity Institute and the Centre for Effective Altruism) and antiutilitarian-leaning groups (e.g., the conservative Anscombe Bioethics Centre and Bioedge.org), as well as groups that focus more on the applied aspects of moral philosophy (e.g., The Hastings Center and Yeoh Tiong Lay Centre). Participants completed the survey online via an electronic link sent in the email. Participants were not personally paid for taking part but instead had the option to register an email address to enter a charity-raffle. For every person that completed the survey we put £2 (approx $2.50 USD) into a pot to be donated to charity, and at the end of data collection we randomly selected one response and asked the winner which charity we should donate to from the following list: Against Malaria Foundation (AMF), Royal Society for the Prevention of Cruelty to Animals (RSPCA); Schistosomiasis Control Initiative (SCI); Cancer Research U.K.; British Heart Foundation; the Red Cross; and Doctors without Borders. The winner chose the Against Malaria Foundation, and we accordingly donated £172 (£2 × 86 participants).

Of the 86 participants who completed the survey in full, five participants were excluded from data analysis because they were undergraduate students. Thus, our final sample consisted of 81 experts in moral philosophy (23 female, *M*_age_ = 32, *SD* = 9.72). Around half of our sample were graduate students (56%), followed by postdoctoral researchers (17%), lecturers/associate professors (14%), and full professors (6%). Participants had spent an average of 8 years (including graduate studies) working in moral philosophy (*M* = 7.65, *SD* = 8.17).

#### Measures

In this study, participants completed the 9-item Oxford Utilitarianism Scale before rating their own self-reported utilitarianism and indicating their own ethical views (see below). Finally, participants indicated their gender and age, their status in the university (graduate student; postdoctoral researcher; lecturer/associate professor; full professor) and how many years they had spent in professional philosophy.

##### Own ethical view

Participants were asked to indicate which of a list of 10 common ethical systems was closest to their own ethical view (act utilitarianism; rule utilitarianism; other form of utilitarianism; nonutilitarian consequentialism; Kantian ethics; other forms of deontology; virtue ethics; care ethics; religious ethics; common-sense morality; non-Western ethical view; or ‘other’). We noted that perhaps none of these would perfectly describe participants’ views, but asked them to indicate which one was the closest.

##### Self-report utilitarianism

To assess how utilitarian our expert participants judged themselves to be, we asked participants to read the following description and rate how utilitarian they were on a 1–10 scale:

We would like you to tell us to what extent you consider your ethical views to be close to or far from utilitarianism. Since ‘utilitarianism’ can mean different things and since there are many ways in which one might reject utilitarianism, let us explain what exactly we mean by this question. By ‘utilitarianism’ we mean unadorned, classical act utilitarianism: roughly, the view that an act is right if and only if it maximizes aggregate welfare from a thoroughly impartial standpoint. So for our purposes views are more utilitarian the closer they are to this view, less utilitarian the further they are from this view. If you are an unqualified act utilitarian then you count as maximally utilitarian on this scale. If you are a rule utilitarian or a consequentialist whose axiology includes more than welfare then you are somewhat less utilitarian. Moving further away from the top end of the scale, the more a person thinks of morality in partial terms, and the more (and the stronger the) deontological constraints they accept, the less ‘utilitarian’ they count on our measure. If you are attracted to W. D. Ross’s pluralist deontological theory you would rank low on this scale. If you hold an absolutist Kantian theory which gives limited weight to consequences, or a highly traditional moral view, then you should rank at the very bottom of the scale. Please indicate where you yourself fall on this dimension, where 10 indicates that you fully endorse classical utilitarianism and 1 indicates that your view is as far as can be from such a utilitarian view. (e.g., you hold very strong deontological views)

### Results

The primary aim of this study was simple: to see whether, for our expert participants who were well-versed in moral philosophy, scores on the OUS would be positively correlated with their own self-reported utilitarianism. Indeed, this is what we found: participants who rated themselves as being more utilitarian on the explicit self-report item also scored higher on the OUS. Participants’ self-reported explicit utilitarianism was significantly correlated with scores on the OUS-IB, *r* = .73, *p* < .001, OUS-IH, *r* = .69, *p* < .001, and overall OUS scores, *r* = .76, *p* < .001. To probe and confirm this primary analysis, we subsequently looked at how participant’s self-reported ethical view (e.g., act utilitarianism, Kantian ethics, etc.) was associated with OUS scores, and conducted analyses controlling for participants’ expertise level. Across all additional analyses the same pattern was observed: self-described utilitarian tendencies were significantly associated with scores on the OUS.

**Figure 1 fig1:**
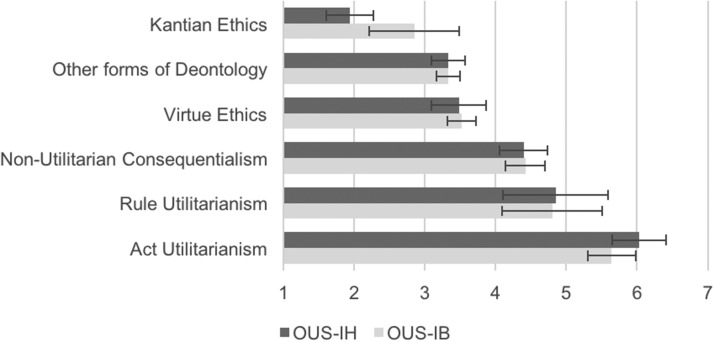
Scores on the OUS as a function of the expert’s self-described ethical view.

First, we looked at OUS scores as a function of the ethical view that our expert participants said characterized their own view (see Figure 1). Participants who described themselves as believing most in “act utilitarianism” (*N* = 11) scored significantly higher on the OUS than those who described themselves as endorsing “other forms of deontology” (*N* = 18): on the OUS-IB, *t*(14.93) = 6.06, *p* < .001, the OUS-IH, *t*(27) = 6.29, *p* < .001, and overall, *t*(14.09) = 6.56, *p* < .001. Similarly, participants who described themselves as some kind of utilitarian (act, rule, or other: *N* = 19) scored higher than everyone else (*N* = 58): on the OUS-IB, *t*(75) = 5.36, *p* < .001, the OUS-IH, *t*(75) = 5.30, *p* < .001, and overall, *t*(75) = 5.81, *p* < .001. And looking just at those people who described themselves as some kind of consequentialist (utilitarian or nonutilitarian: *N* = 36), scores were higher than those endorsing deontological ethics (Kantian or other form of deontology: *N* = 22): on the OUS-IB, *t*(55.68) = 5.83, *p* < .001, the OUS-IH, *t*(56) = 5.10, *p* < .001, and overall, *t*(55.98) = 6.29, *p* < .001. Across the two measures, then, expert participants who reported being more utilitarian scored higher on the OUS. Such results provide strong face validity for our claims: although the OUS is not designed specifically for those with substantial experience with the theory of utilitarianism (one does not need a scale to measure such an expert’s view—you can just ask them!), it was important that differences in the OUS mirrored the self-reported moral views of experts. If a Kantian and a utilitarian scored similarly on the OUS, it would be a rather odd tool with which to assess lay utilitarian tendencies. This, however, was far from being the case. Those who described themselves as utilitarians scored significantly higher on the OUS than those who did not.[Fig-anchor fig1]

Second, we looked at whether results were robust to controlling for years spent in philosophy as a proxy for expertise-level within our expert panel. Partial correlations showed that self-reported explicit utilitarianism was significantly correlated with scores on the OUS when statistically controlling for years spent in philosophy: on the OUS-IB, *r* = .72, *p* < .001, OUS-IH, *r* = .69, *p* < .001, and overall, *r* = .76, *p* < .001. When looking only at graduate students (*N* = 45), self-reported explicit utilitarianism was again significantly correlated with scores on the OUS-IB, *r* = .70, *p* < .001, OUS-IH, *r* = .66, *p* < .001, and overall OUS scores, *r* = .73, *p* < .001. When looking only at those at the postdoctoral level or higher, the same pattern was seen but with slightly higher correlations: on the OUS-IB, *r* = .77, *p* < .001, OUS-IH, *r* = .73, *p* < .001, and overall OUS scores, *r* = .80, *p* < .001. Similarly, when looking at graduates versus postdoctoral researchers or higher, an identical pattern of results was observed when looking at the effects of self-described ethical view on OUS scores.

Turning next to the question of the relation between OUS-IB and OUS-IH, our prediction that these would be more strongly correlated in an expert sample was confirmed (*r* = .75, *p* < .001 in the expert sample compared with *r* = .14, *p* = .02 in the lay sample of Study 2, with these being significantly different, *Z* = 6.5, *p* < .001). This association was not lower in the graduate group (*r* = .74, *p* < .001) compared with the postgraduate one (*r* = .78, *p* < .001; *Z* = −.04, *p* = .68). There was no relationship between years spent in philosophy and scores on the OUS overall, *r* = .14, *p* = .23, the OUS-IB, *r* = .08, *p* = .47, or the OUS-IH, *r* = .17, *p* = .13. Correspondingly, the positive relationship between OUS-IB and OUS-IH was robust to controlling for years spent in philosophy, *r* = .75, *p* < .001.

#### The Oxford Utilitarianism Scale in practice

Having established and validated our new Oxford Utilitarianism Scale, we next looked at how the OUS could be used in practice. Specifically, we sought to explore whether the OUS would shed further light on how utilitarian tendencies are associated with individual differences (e.g., psychopathy), moral attitudes (e.g., prosocial intentions), and ideological factors (e.g., religious belief). In doing so we show that the OUS will be a valuable tool for researchers working in moral psychology, demonstrating how previous work exploring utilitarian tendencies has been limited by a failure to take into account the ways in which the two core aspects of utilitarianism—impartial beneficence and instrumental harm—have separate and divergent influences on moral cognition. By using the OUS we can clarify and extend the relationship between utilitarianism and other constructs.

To look at these issues, we used the same dataset as that used in Study 2 for assessing construct validity in a lay population. As noted above, this dataset consisted of 282 participants recruited from MTurk (178 female, *M*_age_ = 39, *SD* = 12.66), of whom the majority had attended college or higher education (80%). For all of the subsequent discussion, the reader can refer to Table 7 for correlations and to Table 8 for full *M*s and *SD*s. In the Supplemental Materials we report results with other noncentral measures we have omitted here in the interests of space, and further analyses (like those conducted to assess construct validity) looking at patterns of individual differences based on whether the participant was high (i.e., above the scale midpoint) or low (i.e., below the scale midpoint) on both, either, or neither, of the two subscales. Supporting our 2D model, we saw few distinctive patterns of fully utilitarian individuals who scored high on both subscales: for the individual difference measures associated more with the OUS-IB (e.g., empathic concern, identification with all humanity), high-scoring individuals had higher scores on the associated measures, independent of how they scored on the other subscale; and the same pattern was observed for the OUS-IH and the individual differences measures associated with that (e.g., psychopathy).[Table-anchor tbl7][Table-anchor tbl8]

We wish again to highlight before proceeding further that scores on the OUS-IB and OUS-IH were only weakly correlated (Study 1, *r* = .04, *p* = .22; Study 2, *r* = .14, *p* = .02). Moreover, relatively few people in our sample can be characterized as having a genuine overall utilitarian tendency. On the 1–7 scale, the mean overall OUS score was below the midpoint (Study 1, *M* = 3.58, *SD* = 0.86; Study 2, *M* = 3.50, *SD* = 0.92), with slighter higher average scores on the IB subscale (Study 1, *M* = 3.75, *SD* = 1.15; Study 2, *M* = 3.65, *SD* = 1.20) than the IH (Study 1, *M* = 3.37, *SD* = 1.24; Study 2, *M* = 3.31, *SD* = 1.22). Only a quarter of participants (Study 1, 28%; Study 2, 26%) scored above the midpoint of the scale (>4), and this was again higher for the IB (Study 1, 39%; Study 2, 35%) than the IH (Study 1, 27%; Study 2, 24%). This indicates that only a minority of lay people have significant proto-utilitarian leanings. And if we consider a more robust indicator of significant proto-utilitarian tendencies—people who scored at 5 or above on the scale—only 5% in Study 1 and 4% in Study 2 could be classified as significantly utilitarian overall, with this being higher for the IB (Study 1 and Study 2, 16%) than the IH (Study 1, 9%; Study 2, 5%). While there is, then, a distribution of utilitarian tendencies among the population, the prevalence of people who can meaningfully be characterized as having a moderate to strong utilitarian tendency should not be overstated. Befitting its status as a radically demanding and counterintuitive moral philosophy, few laypeople even approximate the views of genuine utilitarians. This is an issue to which we return in our discussion.

#### Personality and individual differences

##### Psychopathy

A growing body of research has shown a relationship between subclinical psychopathy and ‘utilitarian’ judgment in sacrificial dilemmas, whereby ‘utilitarian’ judgments are associated with higher levels of both clinical (Koenigs, Kruepke, Zeier, & Newman, 2012) and subclinical psychopathy and related antisocial traits (Bartels & Pizarro, 2011; Kahane et al., 2015; Koenigs et al., 2012; Wiech et al., 2013). Recent work suggests, however, that this association may be an artifact of the use of sacrificial moral dilemmas as the measure of utilitarianism: although psychopathy is associated positively with a willingness to personally harm others for the greater good, it is associated negatively with endorsement of moral self-sacrifice (Kahane et al., 2015). We predicted, therefore, that although overall OUS scores might be positively associated with psychopathy, this pattern would be driven by the OUS-IH subscale and that there would be either be no relationship, or a negative one, between psychopathy and the OUS-IB subscale. We measured psychopathy using Levenson’s primary psychopathy subscale (Levenson, Kiehl, & Fitzpatrick, 1995), which consists of 16 items including “Success is based on survival of the fittest; I am not concerned about the losers” (α = .89). Supporting predictions, overall OUS scores were not significantly associated with primary psychopathy, *r* = .11, *p* = .07—however, note the *p* value of .07, which could suggest a weak relationship—and neither were scores on the OUS-IB subscale, *r* = .09, *p* = .13. Higher scores on the OUS-IH, however, were significantly associated with increased psychopathy, *r* = .30, *p* < .001, and the strength of correlation between psychopathy and the OUS-IB and OUS-IH was significantly different (*Z* = −2.77, *p* = .01).

##### Empathic concern

What is the relationship between empathic concern and utilitarian tendencies? Some work has suggested that deontological—but not utilitarian—tendencies are related to increased empathic concern (Choe & Min, 2011; Conway & Gawronski, 2013; Gleichgerrcht & Young, 2013; Patil & Silani, 2014). Yet on theoretical grounds, such findings are puzzling, given that empathy has been claimed to be a central psychological source of utilitarianism (Hare, 1981; Smart & Williams, 1973). However, once we take into account that these prior studies all used sacrificial dilemmas as a measure of utilitarianism, the discrepancy can be explained. Such dilemmas focus exclusively on the negative dimension of utilitarianism, and specifically on willingness to cause harm for the greater good. If one considers killing one person to save five others, a stronger empathetic response is likely to be an obstacle to endorsing such instrumental harm. By contrast, the historical tie between utilitarianism and empathy is most plausibly related to utilitarianism’s positive aspect, that is, its impartial concern for everyone’s welfare. It is therefore likely that the previously found negative relationship between empathic concern and utilitarianism may have been driven entirely by instrumental harm, rather than impartial beneficence.

To explore this question we looked at the relationship between scores on the OUS and empathic concern. We used the Empathic Concern subscale of the Interpersonal Reactivity Index (Davies, 1980), which measures sympathy and concern for others and consists of 7 items including “When I see someone being taken advantage of, I feel kind of protective toward them” (α = .90). Given the strong conceptual (and empirical) negative relationship between psychopathy and empathic concern (here: *r* = −.59, *p* < .001), our predictions mirrored those reported above for psychopathy. Specifically, we again predicted that although overall OUS scores might be negatively associated with empathic concern, this pattern would be driven by the OUS-IH subscale and that there would be a positive relationship between empathic concern and the OUS-IB subscale.

There was an overall positive relationship between OUS scores and empathic concern, *r* = .14, *p* = .02, and while both OUS subscales were significantly correlated with empathic concern, these correlations were in opposite directions. While increased empathic concern was associated with scores on the OUS-IB subscale, *r* = .33, *p* < .001, decreased empathic concern was associated with the OUS-IH, *r* = −.16, *p* = .006; these correlations were significantly different (*Z* = 2.28, *p* = .03). Thus, although people who feel greater empathic concern care more about impartially maximizing welfare, they are also less likely to endorse instrumental harm to achieve those ends. In other words, as suggested by Kahane et al. (2015), the negative utilitarian tendency to endorse harm as a means to the greater good might stand in opposition to the positive utilitarian tendency to self-sacrifice and impartially maximizing happiness. Again, at a philosophical level, the aspects of impartial beneficence and instrumental harm are consistent, but at a psychological level these factors appear to have distinct antecedents and consequences on cognition.

##### Identification with all of humanity

We next looked at how OUS scores were associated with scores on the *Identification with All Humanity Scale* (IWAH): a measure of the extent to which people identify with humanity as a whole, rather than exhibit more parochial attachment to one’s own community or country (McFarland, Webb, & Brown, 2012). The extent to which an individual identifies with the whole of humanity is best seen as an affective disposition rather than a moral view. However, such an all-encompassing impartial concern is often claimed to be a core feature of classical utilitarianism (Hare, 1981; Smart, 1956). For this reason, we predicted that IWAH would be positively related to the Impartial Beneficence subscale, but unrelated (or even negatively related) with the Instrumental Harm subscale.

The IWAH Scale was taken from McFarland et al. (2012) and consisted of 9 questions. Participants were required to rate, for people in their community, people in their country, and people all over the world, “How close do you feel to each of the following groups?” In analyzing results, the procedure advised by McFarland et al. was used, regressing the raw scores to give a more accurate representation of the variance in identification with all of humanity, whereby higher scores indicate greater identification with all of humanity.

As predicted, IWAH was significantly associated with OUS scores overall, *r* = .13, *p* = .03, but this was driven in opposite directions by the OUS-IB, *r* = .33, *p* < .001 and OUS-IH, *r* = −.19, *p* < .001, with these correlations being significantly different (*Z* = 6.94, *p* < .001). Therefore, greater impartial beneficence was associated with greater identification with all of humanity, but greater instrumental harm was associated with less identification with all of humanity.

##### Need for cognition

It has been argued that utilitarian decision-making is uniquely based on effortful conscious reasoning (as opposed to immediate emotional responses) and that utilitarian inclinations are uniquely associated with a need for cognition (Conway & Gawronski, 2013), the motivational tendency to engage in effortful thinking (Cacioppo, Petty, & Kao, 1984). Indeed, the tendency for an individual to engage in and enjoy thinking seems intuitively related to utilitarianism to the extent that utilitarian decision-making involves the rational weighing of different consequences and the rejection of simpler, more intuitive solutions to moral problems. It has also often been claimed that greater reliance on reason was the source of the historical emergence of utilitarianism, and generally of more impartial and inclusive moral views (Pinker, 2011; Singer, 2011). The extent to which need for cognition might have divergent relations with the twin factors of impartial beneficence and instrumental harm, though, remains unclear. To explore this question with the OUS, we had participants complete the 18-item Need for Cognition scale (Cacioppo et al., 1984), in which participants rate how characteristic or uncharacteristic certain statements are of them, such as “I would prefer complex to simple problems” (1 = *extremely uncharacteristic of me*; 7 = *extremely characteristic of me*).

In contrast to some previous research, there was no relationship between need for cognition and OUS scores: not overall, *r* = .02, *p* = .73, for the OUS-IB, *r* = .06, *p* = .35 or for the OUS-IH separately, *r* = −.03, *p* = .57. And interestingly, there was no association between need for cognition and other relevant measures, such as Robinson’s utilitarianism scale, *r* = −.09, *p* = .13 or sacrificial moral dilemmas, *r* = −.04, *p* = .46. It is worth noting, however, that need for cognition is a measure of motivation to engage in effortful deliberation. It cannot be ruled out at this stage that the OUS subscales are associated with other indicators of effortful cognition.

#### Moral attitudes

##### Hypothetical donation

Present-day utilitarianism is commonly associated with movements to encourage more charitable donations to strangers in need in developing countries (MacAskill, 2015; Singer, 2015). On utilitarian grounds, donating to effective charities like those in the developing world is not just morally praiseworthy, but actually morally required: we ought to give the money away, and it is wrong not to do so (Singer, 1972). We therefore next looked at how participants’ self-reported tendency to engage in altruistic behavior would be associated with scores on the OUS. To the extent that the OUS measures utilitarianism, it should be positively correlated with a self-reported intention to perform effective charity. To investigate this we used a measure taken from Kahane et al. (2015) where participants were told to:

Imagine that you have just received a bonus at work of $100. Your company, however, has a policy for all staff members that they can either take this bonus money of $100 for themselves with no penalties, or choose to donate it to a respected charity that helps people in the Third World. You can choose to keep or donate as much of the bonus as you wish. If you choose to donate the bonus, the company will double what you put in: if you donate $50 then the company will donate $100, and if you donate $100 the company will donate $200. This choice is made using an anonymized online system, so no one in the company will know what you decided to do.

Participants then rated how much of the bonus they would donate to charity on a scale of $0 to $100. Perhaps unsurprisingly given the importance of charity to contemporary utilitarian thought, overall OUS scores were positively associated with larger hypothetical donations, *r* = .31, *p* < .001. This overall correlation was, however, driven entirely by the Impartial Beneficence subscale, *r* = .40, *p* < .001, with no relationship between hypothetical donations and endorsement of instrumental harm, *r* = .03, *p* = .62. The strengths of these correlations were, again, significantly different (*Z* = 5.00, *p* < .001). It is worth noting that the measure here is strictly a hypothetical one, with no actual cost to participants for making the altruistic decision. And yet people who are higher in the endorsement of instrumental harm still do not even hypothetically donate more. This result would be surprising if utilitarianism reflected a unitary psychological construct, but is to be expected if—as we have argued—what we call utilitarianism actually is grounded in two relatively distinct psychological constructs.

##### Environmental protection

The relationship between utilitarianism and attitudes toward the environment is complex. On the one hand, from a utilitarian perspective natural things such as rivers and rainforests are of only instrumental value, insofar as they bear on the utility of sentient beings. On the other hand, because climate change will likely cause severe harm to humans and other animals, there are strong utilitarian reasons to protect the environment now to prevent that future suffering. We were therefore interested in the relationship between proto-utilitarian tendencies and support for environmental protection. Such support reflects utilitarianism’s positive dimension given that relevant policies require significant self-sacrifice and that most of their beneficiaries will be physically and temporally distant people (indeed, people who will exist only in the future). We thus predicted a positive relation between support for environmental protection and scores on the OUS-IB by contrast, there is no reason to expect such a relation with instrumental harm.

Participants were asked how much they agreed or disagreed that “It is important to protect the environment and the world’s resources because of the negative consequences on future humans if we don’t” (1 = *strongly disagree*, 7 = *strongly agree*). Results showed that there was no overall relationship between OUS scores and support for environmental protection based on utilitarian considerations, *r* = .03, *p* = .67, but this was attributable to the two subscales being significantly associated in different directions. As predicted, increased impartial beneficence was associated with greater endorsement of environmental protection, *r* = .14, *p* = .02, whereas instrumental harm was associated with less willingness to protect the environment, *r* = −.21, *p* < .001; these associations were significantly different (*Z* = 4.55, *p* < .001). Although instrumental harm measures willingness to harm people for the sake of the greater good, it is likely—as suggested by its association with psychopathy—that it also measures general indifference to harm and destruction, including harm to the environment.

#### Ideology

##### Religiosity

How would scores on the OUS relate to religious belief? Religious moral systems often emphasize the importance of rule-based moral decision making. Delve into the holy books of almost all major religions and you will find an extravagant number of rules dictating actions that are either morally obligatory (e.g., observing the Sabbath), or morally impermissible (e.g., coveting thy neighbor’s wife). Such actions are typically seen as morally required or forbidden by virtue of being what they are, rather than as a result of their consequences—God commands it, and so it must be. It is thus not surprising that utilitarianism as a historical movement often came into direct conflict with religious views (Mill, 1863), nor that the utilitarian approach to morality is widely denounced by religious thinkers (Anscombe, 1981; John Paul II, 1995).

In line with the historical relationship between religion and utilitarianism, psychological research has reported that religious individuals are more likely to endorse a nonutilitarian approach to morality (Piazza, 2012; Piazza & Sousa, 2014; Szekely, Opre, & Miu, 2015) and that religious individuals show a tendency to prefer rule-based (deontological) over outcome-based (consequentialist) explanations for the wrongness of a moral action (Piazza, 2012). However the exact nature of the relationship between religious belief and utilitarianism might be less straightforward once we recognize that utilitarianism has both negative (instrumental harm) and positive (impartial beneficence) dimensions. The two-factor OUS therefore makes it possible to investigate the relation between utilitarianism and religious belief in a more fine-grained way than was possible before.

We measured religiosity with a 5-item Centrality of Religiosity Scale (CRS; Huber & Huber, 2012). The CRS is a measure of the centrality, importance, and salience of religiousness in a person, and consists of five items each tapping one of the theoretically defined core dimensions of religiosity: public practice, independent practice, religious experience, ideology, and intellect. Participants were asked to rate on a 5-item scale “How often do you think about religious issues?” (Intellect: 1 = *never*; 5 = *very often*); “To what extent do you believe that God or something divine exists?” (Ideology: 1 = *not at all*, 5 = *very much so*); “How often do you take part in religious services?” (Public practice: 1 = *never*, 5 = *very often*); “How often do you pray?” (Independent practice: 1 = *never*, 5 = *very often*); and “How often do you experience situations in which you have the feeling that God or something divine intervenes in your life?” (Experience: 1 = *never*, 5 = *very often*). Scores were combined into a single reliable measure (α = .93) of the centrality of religiousness for each participant.

Overall OUS scores were associated with religiosity, *r* = .15, *p* = .01, which is perhaps surprising given that previous work has associated deontological thinking with religiosity (Piazza & Sousa, 2014; Szekely et al., 2015). But a positive relation between religiosity and impartial beneficence, that is, the tendency to endorse the impartial promotion of everyone’s welfare even at personal sacrifice to oneself, would be less surprising. Religious systems often include injunctions to “love thy neighbor”—even those from other communities, as exemplified in the parable of the Good Samaritan. Christians are told to “turn the other cheek” and “take up the cross” such that they humble themselves before God and forego material or social benefits in lieu of the divinely ordained moral good. Upon his appointment, Pope Francis said that “It hurts me when I see a priest or a nun with the latest model car; you can’t do this . . . please, choose a more humble one. If you like the fancy one, just think about how many children are dying of hunger in the world” (Francis, 2013). Peter Singer has said almost identical things. Overall, the focus on self-sacrifice and love for one’s neighbors, combined with the idea that all are equal under God’s eyes, might suggest that scores on the OUS-IB would actually be positively associated with religiosity. Indeed, this was what we found, *r* = .15, *p* = .01: those people who were more religious were also those who showed a greater endorsement of the impartial maximization of the greater good. The overall correlation between OUS scores and religiosity thus was driven by the OUS-IB and there was no relationship with religiosity and scores on the OUS-IH, *r* = −.06, *p* = .31; again these were significantly different (*Z* = 2.70, *p* = .01). This pattern of results suggests that the negative relationship observed between utilitarian thinking and religiosity in previous research may be restricted to the component of utilitarianism that rejects traditional deontological moralities, rather than any reduced concern for impartially maximizing welfare per se. Indeed, this contention is supported by a separate analysis with the 6-item Anti-Traditional Morality measure that was not included in the final scale (see above discussion). As would be expected, there was a strong negative relationship such that people who were more religious were less willing to break with the rules of traditional deontological moralities, *r* = −.48, *p* < .001. Given that for our American participants traditional moralities are based heavily in the Judeo-Christian tradition, this result is to be expected.

The positive relationship between religiosity and utilitarianism is not surprising when one considers the dissociation of impartial beneficence and instrumental harm in our 2D model. When Pope John Paul II criticized utilitarianism for treating people as means to an end, he primarily took issue with the instrumental harm aspect of the theory, and indeed it is this focus on instrumental harm that seems to have driven the negative relationship between religiosity and utilitarianism seen among laypeople in previous work. But the positive correlation of religiosity and impartial beneficence is easier to make sense of, reflected in the pronouncements of Pope Francis on the importance of sacrificing material goods for oneself to help those in foreign countries.

Indeed, from a historical perspective, the relationship between utilitarianism and the Judeo-Christian tradition is complex: although utilitarianism has often been historically in conflict with religious belief, some of the direct intellectual precursors of utilitarianism, such William Paley, were Christian believers (Schneewind, 1977); and the self-sacrificing impartial beneficence at the core of utilitarianism has been claimed to have its roots in Christian ideas (Gray, 2015). Sure enough, standard accounts of Christian ethics do generally involve a radical demand for self-sacrifice, impartiality, and universal love. It is really the willingness to harm and the rejection of traditional moral rules that lie at the heart of the historical tension between utilitarianism and religion, and Christian thinkers have recently begun exploring various affinities between the two views (Camosy, 2012).

##### Political ideology

In addition to associating deontological moral thinking with religiosity, some work has suggested that political conservatism exerts a significant effect on deontological moral reasoning—independent of religiosity (Piazza & Sousa, 2014). For this reason, we measured both economic and social political ideology (1 = *very liberal/left*, 7 = *very conservative/right*), as well as asked participants to indicate which political party (if any) they identified with.

Overall OUS scores were not associated with either self-reported economic, *r* = .02, *p* = .69 or social conservatism, *r* = .06, *p* = .28. But, again, more nuanced results were observed when looking at the subscales independently. Economic conservatism was significantly associated with *reduced* impartial beneficence, *r* = −.12, *p* = .054 but *increased* acceptance of instrumental harm, *r* = .18, *p* = .02, with these relationships being significantly different (Z = −3.88, *p* < .001). Such results mesh well with previous work showing an association between libertarianism and prosacrifice judgments in sacrificial dilemmas (Iyer et al., 2012). Social conservatism was not associated with scores on the OUS-IB, *r* = −.06, *p* = .34, but, as with economic issues, greater social conservatism was associated with increased endorsement of instrumental harm, *r* = .18, *p* = .003, and again these associations were significantly different (Z = −3.09, *p* = .002).

A similar pattern of results was seen when looking at political party identification. There was no difference between Republicans and Democrats on overall OUS scores, *t*(194) = −0.35, *p* = .73, but again, this was because party identification was associated with the two subscales in opposite ways. There was a significant difference on OUS-IB scores as a function of political party, *t*(194) = −2.38, *p* = .02, whereby Democrats reported greater impartial beneficence (*M* = 3.77, *SD* = 1.22) than Republicans (*M* = 3.32, *SD* = 1.22). Similarly, there was a significant difference on OUS-IH scores as a function of political party, *t*(194) = 2.34, *p* = .02, but this was such that Republicans reported greater endorsement of instrumental harm (*M* = 3.70, *SD* = 1.21) than Democrats (*M* = 3.25, *SD* = 1.26). In sum, Democrats were more likely to endorse impartial beneficence, and Republicans more likely to endorse instrumental harm. The distinct subcomponents of proto-utilitarian thinking, then, have distinct psychological correlates and even appear to be differently distributed in different groups of people.

#### Demographics

Finally, we looked at the relationship between OUS scores and demographic measures of age, education level, and gender. There was no relationship between age and OUS scores: not overall, *r* = −.06, *p* = .30, for the OUS-IB, *r* = −.08, *p* = .19, or for the OUS-IH, *r* = −.01, *p* = .89. Similarly, there was no relationship between education level and OUS scores: not overall, *r* = −.05, *p* = .40, for the OUS-IB, *r* = −.04, *p* = .56, or for the OUS-IH, *r* = −.04, *p* = .47. There were no gender differences in OUS scores overall, *t*(279) = .04, *p* = .97, nor for the OUS-IB, *t*(279) = −1.40, *p* = .16. While the effect of gender on OUS-IH scores was not significant by conventional standards (see Lakens et al., 2017), *t*(279) = 1.79, *p* = .07, the mean difference was consistent with previous research such that men showed a slightly greater tendency to endorse instrumental harm (*M* = 3.48, *SD* = 1.33) than women (*M* = 3.21, *SD* = 1.13).

## General Discussion

Utilitarianism tells us to impartially maximize the aggregate well-being of everyone—and that we must severely harm or even kill innocent people if doing so is needed to achieve this overarching moral ideal. Most psychological research on utilitarian decision-making has so far focused on this last, negative dimension of utilitarianism, largely ignoring the more foundational impartial ideal that underlies it. In this paper, we have introduced a new theoretical framework for thinking about proto-utilitarian moral thinking in the lay population. Using this framework, we have developed, refined, and validated a new approach to measuring such thinking that taps both positive and negative dimensions of proto-utilitarian tendencies in the lay population. The Oxford Utilitarianism Scale (OUS) is designed to address the limitations of prior measures, especially the currently standard method of measuring so-called ‘utilitarian’ judgments with sacrificial dilemmas. In creating the scale, we took care to ensure that it was conceptually accurate without inappropriately imposing abstract philosophical notions on the moral thinking of nonphilosophers. Thus, although the pool of items we initially considered was based on a thorough analysis of the relevant literature in ethics and vetted by leading professional moral philosophers, the final scale is empirically driven and reflects clusters of moral evaluations that were statistically robust in large samples taken from the lay population. Most importantly, our findings show that proto-utilitarian tendencies do not form a unitary psychological phenomenon in the lay population, and in fact consist of two largely independent subcomponents. This division explains otherwise puzzling results in the existing literature, sheds new light on the psychological sources both of utilitarianism and widespread opposition to it, and opens up new directions for future research.

### The 2D Model of Utilitarian Decision-Making

One of the central ways in which the OUS departs from previous work is by not assuming that utilitarian moral tendencies form a unitary psychological phenomenon, let alone one that can be understood by studying responses in a highly specific moral context. According to our 2D model, utilitarian decision-making involves two dissociable and independently important aspects, measured by separate subscales in the OUS: the first, Impartial Beneficence, reflects the extent to which individuals endorse the impartial promotion of everyone’s welfare, while the second, Instrumental Harm, reflects the extent to which people endorse harm that brings about a greater good. By dissociating these two independent factors of utilitarianism, it is possible to reach a more nuanced and accurate picture of how utilitarian tendencies are related to a host of individual difference measures and other moral attitudes.

The initial results reported here already show how the 2D model of utilitarian decision-making allows us to make better sense of prior findings and associations that would otherwise seem puzzling. They also show how the model can be used to shed light on the psychological sources of the core ‘positive’ aspect of utilitarianism, its radically impartial character and the demands on the self that it consequently imposes. For example, multiple prior studies claimed to find that the moral-decision making of psychopaths is abnormally utilitarian (see, e.g., Koenigs et al., 2012)—a surprising result given the antisocial character of this condition. But once we take into account the distinction between instrumental harm and impartial beneficence as separate dimensions of utilitarian thinking, this result can easily be explained. As reported above, psychopathy in a nonclinical sample was associated with greater endorsement of instrumental harm but not impartial beneficence, a far less surprising result. In a prior study we similarly found a positive relation between psychopathic tendencies and prosacrificial judgments in sacrificial dilemmas but not with the ‘greater good’ dilemmas that pit impartial versus partial moral concerns (Kahane et al., 2015), a finding replicated here.

The 2D model also allows us to better clarify the relationship between utilitarian moral thinking and empathic concern. Again, the negative relationship between utilitarian decision-making and empathic concern suggested by multiple prior studies (Choe & Min, 2011; Conway & Gawronski, 2013; Gleichgerrcht & Young, 2013; Patil & Silani, 2014) is highly puzzling given the historical and, on some views, strong theoretical tie between classical utilitarianism and empathy (Hare, 1981; Smart, 1961/1973) and evidence tying empathic concern to extreme altruism to distant strangers (Brethel-Haurwitz et al., 2016). But our results show that this association is driven exclusively by the instrumental harm dimension of utilitarianism and that empathic concern is at the same time positively associated with impartial beneficence, utilitarianism’s positive core. Indeed, our results show that these two dimensions of utilitarianism are not only independent but are also inversely correlated with a psychological trait that is highly relevant for morality, further confirming our view that it is a mistake to treat utilitarian decision-making as a unitary psychological phenomenon outside of the philosophical context.

In line with interpretation, we also found that while impartial beneficence was associated with greater donation rates to a hypothetical charity helping people in impoverished countries, greater support for welfare-based concern about the environment, and higher levels of identification with the whole of humanity, these measures were either not, or in the case of environmental concern, negatively, associated with instrumental harm.

The 2D model similarly sheds new light on the relation between utilitarian moral thinking and religiosity. Although the two have often been historically opposed, our results suggest that the core of that conflict may lie in utilitarianism’s endorsement of instrumental harm, as well as in its rejection of traditional and absolutist moral rules. Yet religiosity was positively correlated with impartial beneficence, reflecting the important affinities between utilitarianism and Judeo-Christian moral ideals, affinities being explored in some recent work in ethics (Camosy, 2012).

Utilitarianism is typically viewed as a radically progressive moral view. But as the association between impartial beneficence and religiosity reveals, the relationship between utilitarianism and religious and political commitments might be a bit more complicated. This notion is further confirmed by the nuanced relationship between the OUS subscales and political ideologies. For example, economic conservatism was at once positively associated with instrumental harm and, perhaps unsurprisingly, negatively correlated with impartial Beneficence. This finding is in line with previous studies tying libertarianism to prosacrifice judgments in sacrificial dilemmas (Iyer et al., 2012), again illustrating the point that a permissive attitude to harming others is compatible with having a radically partial and even self-centered view of morality (Kahane, 2015). Political party affiliation was also associated with different dimensions of utilitarianism, with Democrats scoring higher on impartial beneficence and Republicans higher on instrumental harm.

Given that the OUS-IB and OUS-IH subscales measure independent psychological factors that are differentially or even inversely associated with a range of traits and measures, it may be questioned whether these two dimensions belong in a single scale. However, the psychological disunity of aspects of utilitarian decision-making in the lay population does not undermine the theoretical unity of the overall construct. The items on this scale were derived from an extensive review of the ethical literature and vetted by a panel of moral philosophers, and scores on both of the subscales were associated with endorsement of an explicit statement of utilitarianism. The two subscales were also strongly correlated in a sample of expert moral philosophers, further confirming their theoretical unity. Finally, ordinary individuals who score highly on both subscales would be appropriately classified as highly utilitarian—and would rank as considerably more utilitarian than someone who merely has a permissive attitude to instrumental harm.

Nevertheless, it is true that focusing on the overall OUS score risks overlooking or even distorting the relationship between utilitarian tendencies and various psychological phenomena. At this early stage, therefore, we suggest that primacy should be given to the subscale scores. Such an approach is not uncommon with measures that target conceptually related but psychologically distinct constructs. A prominent example is the Interpersonal Reactivity Index (Davies, 1980), a multidimensional measure of trait empathy that consists of four independent subscales, each measuring a theoretically related but psychologically distinct trait.

### Instrumental Harm: Implications for the Sacrificial Dilemmas Approach

The sacrificial dilemmas method is currently the dominant approach to measuring utilitarian tendencies (Christensen & Gomila, 2012) and it has yielded important, groundbreaking advances for our understanding of how people consider instrumental harm in moral decision making (Greene, 2014). At the same time, our studies support the argument that sacrificial dilemmas only tap a narrow dimension of utilitarian thinking and that it is unwarranted to infer general claims about the psychological processes underlying utilitarian decision-making only on the basis of the study of prosacrifice judgments in such dilemmas (Kahane et al., 2015; Kahane & Shackel, 2010; Kahane, 2015). Our present work confirms the hypothesis that instrumental harm is a significant dimension of proto-utilitarian tendencies in ordinary people, albeit one that is dissociable from the more positive dimension of impartial beneficence.^8^

The OUS is a measure of individual differences in utilitarian tendencies rather than a tool for directly studying the psychological processes (such as aversion to causing harm) involved in specific episodes of moral decision-making. While much work by Greene and colleagues has focused on how sacrificial dilemmas illuminate the cognitive architecture of moral decision-making rather than utilitarian tendencies per se, sacrificial dilemmas are often used to measure individual differences in utilitarian tendencies within a population as well as differences in such tendencies between populations (see, e.g., Bègue & Laine, 2017; Koenigs et al., 2007). We propose that the OUS should supersede sacrificial dilemmas as a method for measuring such individual differences, even in the domain of instrumental harm. Besides its brevity, the Instrumental Harm subscale of the OUS covers a broader range of considerations relating to instrumental harm than those captured by sacrificial dilemmas, and also avoids the far-fetched and often fantastic character of many sacrificial dilemmas (Bauman et al., 2014).

At the same time, sacrificial dilemmas remain an important tool for studying the psychological processes underlying support for instrumental harm in moral decision-making, one aspect of utilitarian decision-making. Notice, however, that while the OUS was designed to measure the moral outlooks of *individuals* rather than the processes underlying moral *judgments*, it can also shed light on the considerations that shape moral judgments in more specific contexts. Indeed the OUS could be used to distinguish those prosacrifice judgments that reflect only greater acceptance of instrumental harm and those that may also reflect a broader impartial moral perspective; the findings reported here suggest that there may be such a subset of prosacrifice responses to sacrificial dilemmas given that such responses were associated with impartial beneficence (albeit weakly).

All of that said, we believe that there is a strong case for abandoning the widespread but unhelpful terminological practice of classifying prosacrifice responses in sacrificial dilemmas as ‘utilitarian judgments’ (Kahane, 2015; Kahane et al., 2015). On that terminology, a psychopath who is concerned only about his self-interest and has no compunction about harming others would be described as having a ‘strong utilitarian bias’ while an extreme altruist who is a moral vegetarian, gives most of her money to charities helping people in the developing world, and who donated her kidney to a stranger, yet who recoils from the idea of violently sacrificing an innocent person to save five others, would count as having a ‘strong deontological bias.’ By contrast, the OUS would reveal that these two individuals are unusually high on one aspect of a utilitarian outlook but low on the other. Neither is usefully described as fully utilitarian, although it could be argued that the extreme altruist comes much closer.

While prosacrifice judgments are in line with utilitarianism, so are many other moral judgments, including, most paradigmatically, judgments relating to self-sacrifice and aid to strangers—what we call the positive dimension of utilitarianism. Reserving the term ‘utilitarian judgment’ to the narrow, negative aspect of a utilitarian outlooks leaves us with no terminology to describe these other classes of judgments, and gives the misleading impression that instrumental harm is the core or even the whole of utilitarianism. When researchers discuss the psychological factors and processes that underlie ‘utilitarian judgments’ they can give the impression that they are referring to a general, unitary psychological phenomenon. Yet there is little or no evidence that the processes that drive prosacrifice judgments (reflecting greater endorsement of instrumental harm) also drive, for example, judgments instructing us to give a substantial portion of our income to help distant strangers. In fact, the findings of the present study and of Kahane et al. (2015) strongly suggest that very different processes shape these different kinds of proto-utilitarian judgments, and that some factors—most notably, empathic concern—may play a different and even contrasting role in each case.

Theoretical clarity and precision would be better served, for example, if instead of describing the prosacrificial tendencies of individuals with antisocial traits as a ‘utilitarian bias,’ it was instead referred to as a ‘consequentialist bias’ (since consequentialism need not imply the positive, impartial aspect of utilitarianism), or even more precisely as a bias in favor of instrumental harm. Importantly, on our framework such individuals *are* more utilitarian than others in one important respect, even if not in others. Even if a bias in favor of prosacrificial judgments is strongly driven by reduced aversion to harm, its overt manifestation seems to be genuinely focused on what we called Instrumental Harm—that is, seeing harm as permitted only when it leads to a morally better consequence. This interpretation would be defeated only if such individuals had a morally permissive attitude toward harming irrespective of consequence. Yet this seems unlikely, at least in most individuals whose antisocial tendencies are subclinical.

### Impartial Beneficence: Preliminary Findings and Future Directions

Although the psychology of instrumental harm has received a great deal of attention in moral psychology, less work has been devoted to investigating the sources of radically impartial moral views. There is of course a considerable literature studying altruism (e.g., Batson, 1991; de Waal, 2008; Krebs, 1982) and charitable giving more specifically (e.g., Bekkers & Wiepking, 2011; Caviola, Faulmüller, Everett, Savulescu, & Kahane, 2014; Harbaugh, Mayr, & Burghart, 2007; Oppenheimer & Olivola, 2011; Van Lange, Bekkers, Schuyt, & Vugt, 2007), but as discussed earlier, impartial beneficence goes beyond a willingness to make sacrifices to help others, or even a willingness to make sacrifices to help complete strangers who will not reciprocate (as studied, e.g., using the Dictator Game; see, e.g., Benenson, Pascoe, & Radmore, 2007; Brañas-Garza, 2006; Eckel & Grossman, 1996; Engel, 2011; Everett, Haque, & Rand, 2016). Individuals differ in the degree to which they are disposed to make sacrifices to help strangers, but such differences are compatible with commonsense morality since it regards such sacrifices as permissible and even praiseworthy. Impartial beneficence, by contrast, is the far more radical view that we should treat the well-being of *all* sentient beings *equally*—and that we are therefore *required* to give as much moral weight to distant strangers as to our closest relatives. Although such impartial beneficence is theoretically distinct from identification with the whole of humanity (McFarland et al., 2012), a construct more concerned with an individual’s sense of identity, the two constructs are obviously closely related and as expected were strongly correlated in our research.^9^

Impartial beneficence was also associated with greater levels of empathic concern, suggesting an important role for emotion in the core positive dimension of utilitarianism. This finding is in line with the theorizing of some prominent utilitarians (Hare, 1981; Smart & Williams, 1973), but in tension both with accounts of impartial beneficence that see it as based in cold reason (de Lazari-Radek & Singer, 2012; Sidgwick, 1901) and some recent psychological theorizing (Greene, 2008). The link with empathic concern is also consonant with work suggesting that the psychological and neural profile of extreme altruists is the reverse of that of psychopaths (Marsh et al., 2014). To the extent that extreme altruism is driven by unusual levels of empathic concern (Brethel-Haurwitz et al., 2016), the 2D model predicts that such extreme altruists would be high in impartial beneficence but low on instrumental harm. And although we did not find a negative association between psychopathy and impartial beneficence in the lay population, we have previously found that psychopathy was linked to reduced endorsement of self-sacrifice for the greater good in the ‘greater good’ vignettes (Kahane et al., 2015).

While impartial beneficence is not identical with self-sacrifice, even in its extreme forms, it is still the case that 3 of the 5 items in the OUS-IB focus on self-sacrifice. Further research is needed to clarify the relationship between endorsement of self-sacrifice and refusal to give priority to those who are emotionally, socially, spatially, and temporally near—two aspects of impartiality that are potentially distinct. More generally, just as work using sacrificial dilemmas has revealed a highly complex picture of the multiple factors that can shape judgments relating to instrumental harm, it is likely that a wide range of factors can affect judgments relating to impartial beneficence. The ‘greater good’ vignettes introduced in Kahane et al. (2015), which cover different aspects of partiality/impartiality, could be used to study such factors.

The association between impartial beneficence and empathic concern may seem inconsistent with recent criticisms of empathy that contrast it with a more impartial, consequentialist approach (Bloom, 2017). However, that criticism targets empathy understood as feeling what you believe others feel, and as contrasted with compassion or concern for the well-being of others (Jordan, Amir, & Bloom, 2016): it is largely the latter which is measured by the empathic concern subscale employed in the present study (Davies, 1980). Still, further research into the relationship between empathic concern and different aspects of moral impartiality is needed. Moreover, it should not be assumed that an attitude of impartial beneficence will be automatically translated into focused efforts to maximize utility of the kind advocated by the effective altruist movement (MacAskill, 2015; Singer, 2015). It may be that a tendency toward impartial beneficence makes up the affective background to such an explicit maximizing aim, but that further cognitive steps are needed to endorse it explicitly.

Recent work in moral psychology and its evolutionary origins has highlighted the ‘groupish’ character of human morality (Greene, 2014; Haidt, 2012) and the ways in which altruism is often constrained by self-interest (e.g., Bardsley, 2008; Dana, Cain, & Dawes, 2006; Yamagishi & Kiyonari, 2000) and social distance (Jones & Rachlin, 2006). Commonsense morality allows individuals to prioritize self over others, family and friends over strangers, and country and other ingroups over outgroup members. The British Prime Minister Theresa May recently stated that “If you believe you’re a citizen of the world, you’re a citizen of nowhere.” Further familiar injunctions include “Family comes first” and “Charity begins at home.” The impartial cosmopolitan outlook of utilitarianism is a radical departure from such commonsense attitudes. But although the factors that underlie allegiance to various ingroups have been studied extensively, the psychological underpinnings of such an impartial moral view have received less attention.

Interestingly, recent work has found that participants expressed negative attitudes toward actors who sacrificed one to save a larger number in sacrificial dilemmas (Everett, Pizarro, & Crockett, 2016) as well as toward actors who acted impartially by not giving priority to those with whom they have close personal relationships (Hughes, 2017). It is not surprising that both aspects of utilitarianism are regarded with suspicion—the utilitarian ideal of impartial beneficence was already ridiculed by Charles Dickens in *Bleak House* in his character of Mrs. Jellyby, a ‘telescopic philanthropist’ who is obsessed with aiding a remote African tribe while showing little concern for her own family (Dickens, 1853). At the same time, such an impartial attitude is unlikely to arouse the aversive reaction most people feel toward the idea of violently sacrificing an innocent person—let alone toward the idea of infanticide. Moreover, Hughes (2017) found that impartial acts were regarded as less moral than partial ones because of inferences about lack of empathy and compassion; yet we found that impartial beneficence is closely tied to empathic concern. In addition, Hughes (2017) focused on cases where actors do not give priority to those close to them. Attitudes toward actors who engage in acts of extreme self-sacrifice are likely to be more ambivalent, and may be influenced by whether such acts are regarded by the actor as supererogatory (i.e., going beyond the call of duty) or, in line with utilitarianism, as obligatory—and thus as issuing a similar demand to others.

The evidence currently suggests that the variance in moral views about instrumental harm is strongly driven by differences in aversion to harming. Individuals who feel a strong aversion to harming tend to reject Instrumental Harm; those with less or no such aversion regard it more permissively. There is at present less evidence about the sources of differences in impartial beneficence. It is very likely that low OUS-IB scores, indicating strong partiality, reflect either a strongly self-centered outlook and reluctance to make significant sacrifices to others, or a strong commitment to various narrow ingroups of the kind measured, for example, by the loyalty subscaled of the Moral Foundations Questionnaire (Graham et al., 2011). But although factors driving such strongly partial views are well-studied, the factors that drive above-average impartial views, all the way to the radical impartiality of utilitarianism, are not as well understood. For example, whereas within a utilitarian framework there is no inherent difference between the rejection of partiality to self and a rejecting of partiality to close others, these two aspects of radical impartiality may have distinct psychological sources in the lay population.

Because the present study only investigated the correlates of impartial beneficence, it cannot speak directly to the question of what causally drives it. The tie we found between impartial beneficence and empathic concern, and the absence of an association with need for cognition, is in line with the notion that such radical impartiality is driven by empathic concern, an affective disposition, broadly in line with some philosophical accounts of the sources of utilitarianism (Hare, 1981; Smart, 1961/1973) but less so with the view that such impartiality is based on rational reflection on the arbitrariness of privileging some humans over others or of giving moral significance to mere distance (Sidgwick, 1907; de Lazari-Radek & Singer, 2012). However, further work is needed to directly investigate this question.

Although impartial beneficence and instrumental harm are two independent dimensions of moral judgment, they can interact in important ways. Views on impartial beneficence determine which beings are taken to fall within the scope of morality and the extent to which they are given moral weight. These considerations will in turn affect judgments about whether certain people (who may be family members, compatriots, or strangers) should be sacrificed to save others (who again may be close or distant from the agent; see Petrinovich et al., 1993; Swann et al., 2010). Conversely, views on instrumental harm will affect how one will approach promoting the greater good: are we permitted to promote this impartial aim by engaging in cold calculation of cost and benefit, sacrificing some to help a greater number, or must our efforts to help others also respect the rights of individuals? This two-way relationship between the two factors offers further justification to measuring both of them in a single scale.

### The Psychological Sources of Utilitarianism and Anti-Utilitarianism

Utilitarianism is often portrayed by its proponents as the product of clear-headed rational reflection, and resistance to utilitarianism as largely due to the way it clashes with powerful intuitions and emotions (such as those elicited by the idea of violently sacrificing an innocent person to maximize utility; Singer, 1974, 2005). Greene (2008) has argued that rather than deontology and consequentialism being “philosophical inventions,” they are better understood as “philosophical manifestations of two dissociable psychological patterns, two different ways of moral thinking, that have been part of the human repertoire for thousands of years” (p. 360). We concur that support for utilitarian (consequentialist) or deontological ethical principles is almost certainly driven by psychological dispositions and processes that are part of our basic mental toolkit. Where we disagree is with the notion that the utilitarian side of the equation is the result of a single, unitary psychological pattern. This is because our findings suggest that there are two dissociable psychological sources *within* the utilitarian dimension: one relating to impartial beneficence and one to instrumental harm. In other words, our 2D model posits that there are two dissociable components to utilitarian decision-making—something quite different from Greene’s dual process model, which posits two systems underlying deontology versus utilitarianism, while treating each as a unitary psychological construct. On Greene’s model, that is, the ‘dual’ refers to automatic versus controlled processes underlying deontological versus utilitarian judgments respectively; in our model, the ‘two’ refers to two different components within utilitarianism.

While a unitary model of utilitarianism-as-cognitive (and deontology-as-emotional) seemed to be supported by earlier studies using sacrificial dilemmas that tied prosacrifice judgments to effortful deliberation (Greene et al., 2004), more recent work has related such judgments to reduced aversion to harming (Cushman, Gray, Gaffey, & Mendes, 2012; Kahane et al., 2015). This latter work suggests therefore that prosacrifice judgments are largely driven by reduced emotion. In line with this interpretation, in the present study we found an association between the Instrumental Harm subscale and psychopathy and reduced empathic concern. The positive dimension of utilitarianism has also often been claimed to be based in rational reflection (de Lazari-Radek & Singer, 2012; Sidgwick, 1907)—Peter Singer’s (1972) famous argument that there is no moral difference between letting a drowning child die and refusing to donate money to prevent deaths in developing countries is based on an appeal to consistency, not on pulling at our heartstrings. As we saw, however, the present study also associates impartial beneficence with empathic concern, an affective disposition—indeed, one that is exactly the reverse of that associated with instrumental harm. At the same time, neither subscale was significantly associated with need for cognition, a trait measure of motivation to engage in effortful cognition. These results are consonant with recent studies that found that extreme altruists who donated their kidneys to strangers exhibit higher empathic concern (Brethel-Haurwitz et al., 2016).

Importantly, our 2D model suggests a hitherto overlooked source of opposition to utilitarianism. Utilitarianism notoriously has implications that many find highly counterintuitive and even repugnant. Individuals low in both instrumental harm and impartial beneficence are likely to be strongly resistant to utilitarianism. However, the psychological independence and degree of tension between these two dimensions of utilitarianism suggests a further obstacle to its acceptance: psychological factors such as empathic concern that may make some individuals receptive to one dimension may also make them resistant to the other. There might be a degree of psychological instability *internal* to utilitarianism. As noted above, however, further research is required to clarify the causal relationship between empathic concern and impartial beneficence.

Instrumental harm and impartial beneficence are two aspects of a single coherent philosophical theory, but they come apart in the psychology of ordinary people. It is striking that while the two were only weakly associated in the lay population, they were strongly correlated in a sample of expert moral philosophers. It is unclear what explains this sharp contrast, and at this stage we can only speculate about its source. One natural explanation would be that this change may be due to philosophical education, including exposure to explicit views and arguments that tie the two moral dimensions together. On the 2D model, individuals are likely to arrive at such an overall utilitarian view by following two distinct psychological paths. Some individuals—perhaps driven by unusually high levels of empathic concern—begin by endorsing a radically impartial vision of moral concern and, in an attempt to turn this endorsement into a coherent theory, eventually come to endorse forms of instrumental harm as well, to promote such impartial goals. Other individuals may start with greater acceptance of instrumental harm—likely driven by low levels of empathic concern—and a general rejection of traditional moral rules, and, seeking to find a systematic moral framework to replace the commonsense morality that they reject, come to endorse a sweeping impartial view. In both cases, reasoning may serve not as the impetus to the embryonic utilitarian view, but rather as a means to integrate two aspects of utilitarianism that are psychologically independent or even opposed. Utilitarianism may be the product, not of pure rational reflection and argument, but of an attempt to bring pretheoretical tendencies and intuitions into a coherent equilibrium (along the lines suggested, in the deontological context, by Holyoak & Powell, 2016). Further research could investigate (a) the extent to which explicit endorsement of utilitarian views involves such adjustment, (b) whether one of the two ‘starting points’ is predominant, and (c) whether the initially dominant dimension predicts the degree to which the behavior of utilitarians mirrors their theoretical commitments. One can predict, for example, that individuals who start out high only in instrumental harm give less money to charity compared with those who start out high in impartial beneficence.^10^

An alternative explanation of the association between the two subscales in the expert sample is that the structure of the debate in current moral philosophy attracts those individuals in whom the two dimensions are already aligned. Possible support for this speculative hypothesis comes from the fact that the proportion of the lay population that could be described as having strong overall utilitarian tendencies—around 26% scored above the midpoint of the OUS (>4) and only 4% scored 5 or more—is not substantially lower than the self-reported view of philosophers. Of 931 participants in a recent survey of professional philosophers, 23.6% favored or leaned toward consequentialism over deontology or virtue ethics; of these, only 9.7% endorsed consequentialism outright (Bourget & Chalmers, 2014). Because consequentialism includes both utilitarianism in its different forms as well as theories that ascribe value to things other than utility (e.g., to justice), these results suggest that the percentage of professional philosophers who endorse utilitarianism without qualification may not be much higher than the percentage of participants in our study who consistently endorsed strongly utilitarian views. Coupled with our own findings, this at least raises the possibility—a possibility that requires further investigation—that full-blown endorsement of utilitarianism partly reflects an unusual prephilosophical psychological disposition in a minority of individuals, rather the end point of a process of philosophical reasoning from a common psychological starting point shared by nonutilitarians. A simpler alternative explanation, however, is that utilitarian views are endorsed only by a small minority both in moral philosophy and in the general population because of the general cultural dominance of deontological forms of morality.

### Limitations and Future Directions

The OUS does have a number of limitations. Some of these have been mentioned in passing in the discussion above but it is worth making them explicit. First, the OUS is a measure of individual differences in moral outlook, not of the processes and mechanisms that underlie a particular episode of moral judgment; it measures traits rather than states. Second, the items on the OUS-IB largely focus on self-sacrifice and less on impartiality with respect to others: a more fine-grained approach may be needed to investigate different strands of the psychology of moral partiality and impartiality.

A third potential limitation of the OUS is that it doesn’t directly measure the calculating dimension of utilitarianism. Classical utilitarians not only claim that we should impartially consider the good of all in our moral decisions—they also hold that we are morally required to *maximize* that good, and it is this calculating and maximizing aspect of utilitarianism that some find problematic. Many items on both subscales of the OUS implicitly involve comparisons of overall utility (sacrificing one’s leg to save another’s life; killing some innocent people to save a greater number), and the OUS was positively associated with an explicit statement of utilitarianism that included this maximizing component. But the scale admittedly does not directly measure this dimension of utilitarianism. This is for two reasons. First, to directly measure such a maximizing tendency, we would need to see whether subjects would endorse, not only sacrificing 1 to save 5, but 1 to save 2, and 20 to save 21 (Kahane, 2015). A short item scale is not the best tool for this achieving this end. Second, it is doubtful whether such a maximizing tendency is a distinctive moral phenomenon rather than a general decision-making tendency—an egoist driven by pure self-interest can also engage in elaborate cost-benefit analysis when others may merely satisfice. We concede, however, that the question of whether such a maximizing tendency reflects a distinctive dimension of moral decision-making and, possibly, even a further dimension of proto-utilitarian tendencies, is worth investigating.^11^

Finally, we wish to emphasize that the OUS is meant to be a measure of an overall pattern of moral views and judgments, not behavior or intentions to act. Individuals may strongly endorse instrumental harm yet find it difficult to sacrifice someone to save a greater number if actually confronted with such a decision in real life. Similarly, someone may endorse a highly impartial vision of morality yet fail, for example, to donate much money to relevant effective charities. Indeed, several studies found that the moral behavior of moral philosophers does not significantly differ from that of others (Schwitzgebel, 2009; Schwitzgebel & Rust, 2009, 2014). We did find that impartial beneficence was positively associated with greater rates of hypothetical donation to charity but further research is needed to clarify the relationship between the subscales of the OUS and relevant forms of actual moral behavior.

### Conclusion

The use of sacrificial dilemmas to study the contrast between utilitarian and deontological judgments has dominated research in moral psychology. But although the psychological source of this major ethical dispute is of great interest, sacrificial dilemmas are a limited tool for studying proto-utilitarian tendencies in the lay population (Kahane, 2015; Kahane et al., 2015; Kahane & Shackel, 2010). In this paper we have introduced the 2D model of utilitarian psychology, a new theoretical framework for studying such tendencies. The 2D model treats utilitarian moral decision-making not as an all-or-nothing category but as a matter of degree, and as involving two largely independent ‘positive’ and ‘negative’ dimensions. On this basis, we developed the OUS, a new scale that is both philosophically rigorous and empirically driven, and which attempts to address concerns about sacrificial dilemmas and existing scales. Our preliminary application of the scale already demonstrates how the distinction between impartial beneficence and instrumental harm can help to clarify the relationship between a utilitarian moral outlook and a range of other psychological constructs and measures while generating important new avenues for further research. Importantly, our results strongly suggest that, in the context of the lay population, utilitarian decision-making does not constitute a unitary psychological phenomenon.

The division between impartial beneficence and instrumental harm may also have important practical implications. Ethicists who wish to promote wider acceptance of utilitarian moral approaches in the general population may need to divide their efforts. Those individuals who are more likely to endorse instrumental harm, or generally be willing to dismiss or discount traditional moral rules, may at the same time be indifferent—or even hostile—to the overarching moral aim of impartially maximizing the good of all sentient beings. Conversely, drawing public attention to the negative side of utilitarianism—one upshot of the widespread identification of utilitarianism with sacrificial solutions to trolley dilemmas in current moral psychology—may do little for, and even get in the way of, promoting greater moral impartiality. Singer’s session on effective altruism at Victoria University drew those who were excited by the idea of impartial beneficence—but also a group of outraged protestors repelled by instrumental harm. To the extent that the positive aim of utilitarianism has greater moral priority, utilitarians would be advised to downplay the negative component of their doctrine and may even find a surprisingly pliant audience in the religious population.

But although such a strategy may be a more effective way of promoting aspects of utilitarian thinking, the apparent psychological disconnect between the ‘positive’ and ‘negative’ dimensions of utilitarianism—an attitude of impartial concern for the well-being of all individuals, on the one hand, and a ‘cold-hearted,’ unemotional acceptance of instrumental harm on the other—suggests that the prospect of full-blown, unreserved acceptance of utilitarianism by more than a small minority may face formidable psychological obstacles.

## Supplementary Material

10.1037/rev0000093.supp

## Figures and Tables

**Table 1 tbl1:** Factor Loadings for the 10th EFA Resultant Rotated Factor Matrix

Item	Factor loadings			
	Factor 1	Factor 2	Factor 3	Factor 4
14	.74	—	—	—
16	.65	—	—	—
17	.65	—	—	—
11	.61	—	—	—
15	.48	—	—	—
62	.48	—	—	—
77	.48	—	—	—
73	.47	—	—	—
61	.43	—	—	—
63	.43	—	—	—
48	.41	—	—	—
68	—	.77	—	—
69	—	.76	—	—
67	—	.68	—	—
72	—	.67	—	—
20	—	.48	—	—
59	—	.44	—	—
70	—	.37	—	—
52	—	.36	—	—
43	—	.35	—	—
26	—	—	.70	—
27	—	—	.63	—
2	—	—	.62	—
25	—	—	.59	—
18	—	—	.54	—
4	—	—	.54	.30
57	—	—	.43	—
28	—	—	.38	—
21	—	—	.34	—
23	—	—	.34	—
44	—	—	—	.76
47	—	—	—	.72
3	—	—	—	.64
45	—	—	—	.61
46	—	—	—	.44
Alphas	81	79	79	79
*Note*. — indicates that the factor loading was < .30. EFA = exploratory factor analyses.				

**Table 2 tbl2:** Item Labels for Final Items Used in CFA (Study 1)

Item	Factor	Item label	Final item and subscale
2	3	There are some things that are simply right or wrong, no matter what the consequences.	
3	4	It is sometimes acceptable to break a moral rule in order to do good.	
4	3	Some moral rules should never be broken, no matter how good the consequences.	
11	1	From a moral point of view, we should feel obliged to give one of our kidneys to a person with kidney failure since we don’t need two kidneys to survive, but really only one to be healthy.	IB-1
14	1	We have a moral obligation to do everything we can to help others in need, even if this requires giving away most of our money.	
15	1	From a moral point of view, we shouldn’t act in self-defense if this would cause great harm to other innocent people.	
16	1	It is morally wrong to keep money that one doesn’t really need if one can donate it to causes that provide effective help to those who will benefit a great deal.	IB-2
17	1	If the only way to save another person’s life during an emergency is to sacrifice one’s own leg, then one is morally required to make this sacrifice.	IB-3
18	3	Some of society’s laws and rules should never be broken.	
20	2	If the only way to ensure the overall well-being and happiness of the people is through the use of political oppression for a short, limited period, then political oppression should be used.	IH-2
21	3	Sometimes it’s inevitable that something morally bad will occur, but it’s much worse if you’re the one who made it happen.	
23	3	Virtues like kindness and wisdom are morally important in and of themselves, whether or not they lead to good consequences overall.	
25	3	Some things are wrong because they violate human dignity, even if they would lead to better consequences.	
26	3	Some things are wrong because they are contrary to nature, even if no one is harmed.	
27	3	Some sexual acts and relationships are inherently wrong, even if they are consensual and nobody is harmed.	
28	3	If someone is in a position of legitimate authority over us, we have a moral duty to do and respect what they say, even if we personally think this will lead to a worse result.	
43	2	Freedom has to be weighed against the public welfare: if it is necessary to restrict individuals’ freedom to promote the greater good, then that is what should be done.	
44	4	It is morally permissible to lie if doing so would help others a great deal.	
45	4	It is morally wrong to lie to a person, even if it is for their own good, and will make them better off.	
46	4	It is morally wrong to break promises even if this would bring about good outcomes.	
47	4	It is important to be truthful as a general rule, but sometimes people have to lie to do the right thing.	
48	1	It is morally wrong for you to take a high-paying job in an industry that causes some harm (such as tobacco or petrochemical)—even if you would donate much of your earnings to an important charity that would prevent a greater amount of harm.	
52	2	If faced with the choice, it is morally better to kill a human being who is severely mentally disabled than it is to kill a healthy chimpanzee with greater self-awareness and cognitive and emotional capacities.	IH-3
57	3	Criminals should receive the punishment they deserve—even if this will not protect the public or deter crime in the future.	
59	2	If letting an innocent person go free in some particular instance would certainly cause riots—riots that would lead to a serious loss of life—then it is OK to send this innocent person to jail.	
61	1	If someone had a choice between saving the life of their own child, versus saving the lives of three strangers’ children, it would be morally better for that person to save the three strangers’ children.	
62	1	From a moral perspective, people should care about the well-being of all human beings on the planet equally; they should not favor the well-being of people who are especially close to them either physically or emotionally.	IB-5
63	1	When trying to help suffering people, it is just as morally important to help those in a faraway country as it is to help those suffering in your own neighborhood.	
67	2	Sometimes it is morally necessary for innocent people to die as collateral damage—if more people are saved overall.	IH-5
68	2	It is morally right to harm an innocent person if harming them is a necessary means to helping several other innocent people.	IH-1
69	2	It is morally right to harm a single innocent person if harming them is a necessary means to preventing harm to a greater number of innocent people.	
70	2	It is morally wrong to take advantage of someone, even if it is to help a large number of other people.	
72	2	It is permissible to torture an innocent person if this would be necessary to provide information to prevent a bomb going off that would kill hundreds of people.	IH-4
73	1	It is just as wrong to fail to help someone as it is to actively harm them yourself.	IB-4
77	1	Failing to send money to a charity to save an innocent person’s life—someone you know will die without your help—is just as morally bad an action as sending a package of poison that will directly cause that person to die.	
*Note*. IB = Impartial Beneficence subscale; IH = Instrumental Harm; CFA = confirmatory factor analysis.			

**Table 3 tbl3:** Recommended and Actual Model Fit Indices

Measure	Recommended value	Study 1			Study 2		
		Factor 1 OUS-IB	Factor 2 OUS-IH	2-factor solution Overall OUS	Factor 1 OUS-IB	Factor 2 OUS-IH	2-factor solution Overall OUS
RMSEA	≤.07	.07	.04	.05	.07	.05	.04
SRMR	≤.08	.03	.01	.04	.03	.02	.04
CFI	≥.95	.97	.10	.97	.96	.99	.98
*Note*. RMSEA = root mean square error of approximation; SRMR = standardized root-mean-square residual; CFI = comparative fit index; OUS-IB = Impartial Beneficence Sub-Scale; OUS-IH = Instrumental Harm Sub-Scale; OUS = Oxford Utilitarianism Scale.							

**Table 4 tbl4:** Factor Loadings for the Final Two-Factor CFAs in Studies 1 and 2

Item	Study 1		Study 2	
	Factor 1 “Impartial Beneficence”	Factor 2 “Instrumental Harm”	Factor 1 “Impartial Beneficence”	Factor 2 “Instrumental Harm”
62	.44		.38	
11	.78		.74	
17	.71		.68	
73	.47		.50	
16	.56		.57	
20		.43		.45
68		.72		.78
72		.68		.67
67		.79		.73
*Note*. All factor loadings are significant at the *p* < .001 level; CFA = confirmatory factor analysis.				

**Table 5 tbl5:** Final Items and Item Numbers for the Oxford Utilitarianism Scale

Subscale	Development item No. (Study 1)	Final item No.	Final item
Impartial Beneficence	62	IB-1	From a moral perspective, people should care about the well-being of all human beings on the planet equally; they should not favor the well-being of people who are especially close to them either physically or emotionally.
	11	IB-2	From a moral point of view, we should feel obliged to give one of our kidneys to a person with kidney failure since we don’t need two kidneys to survive, but really only one to be healthy.
	17	IB-3	If the only way to save another person’s life during an emergency is to sacrifice one’s own leg, then one is morally required to make this sacrifice.
	73	IB-4	It is just as wrong to fail to help someone as it is to actively harm them yourself.
	16	IB-5	It is morally wrong to keep money that one doesn’t really need if one can donate it to causes that provide effective help to those who will benefit a great deal.
Instrumental Harm	68	IH-1	It is morally right to harm an innocent person if harming them is a necessary means to helping several other innocent people.
	20	IH-2	If the only way to ensure the overall well-being and happiness of the people is through the use of political oppression for a short, limited period, then political oppression should be used.
	72	IH-3	It is permissible to torture an innocent person if this would be necessary to provide information to prevent a bomb going off that would kill hundreds of people.
	67	IH-4	Sometimes it is morally necessary for innocent people to die as collateral damage—if more people are saved overall.
*Note*. IB = Impartial Beneficence subscale; IH = Instrumental Harm.			

**Table 6 tbl6:** Correlations Between the OUS and Other Measures of Utilitarianism

Measure	1	2	3
1. Overall Oxford Utilitarianism Scale (OUS)	—		
2. Impartial Beneficence Sub-Scale (OUS-IB)	.81**	—	
3. Instrumental Harm Sub-Scale (OUS-IH)	.70**	.14*	—
4. Explicit utilitarianism	.35**	.37**	.13*
5. Classic sacrificial dilemmas	−.34**	−.21**	−.32**
6. Greater good dilemmas	.40**	.50**	.07**
* *p* < .01. ** *p <* .005.			

**Table 7 tbl7:** Correlations Between the OUS and Related Individual Differences Measures

Measure	1	2	3
1. Overall Oxford Utilitarianism Scale (OUS)	—		
2. Impartial Beneficence Sub-Scale (OUS-IB)	.81**	—	
3. Instrumental Harm Sub-Scale (OUS-IH)	.70**	.14*	—
4. Psychopathy	.11	−.09	.30**
5. Empathic concern	.14*	.33**	−.16**
6. Identification with all of humanity	.13**	.33**	−.19**
7. Need for cognition	.02	.06	−.03
7. Hypothetical donation	.31**	.40**	.03
8. Environmental protection	−.03	.14*	−.21**
9. Economic conservatism	.02	−.12	.18**
10. Social conservatism	.06	−.06	.18**
11. Religiosity	.15*	.15*	.06
* *p* < .01. ** *p* < .005.			

**Table 8 tbl8:** Ms and SDs for Measures in Study 2

Measure	Scale rating	*M*	*SD*
Overall Oxford Utilitarianism Scale (OUS)	1–7	3.50	.92
Impartial Beneficence Sub-Scale (OUS-IB)	1–7	3.65	1.20
Instrumental Harm Sub-Scale (OUS-IH)	1–7	3.31	1.22
Explicit utilitarianism	1–5	2.99	1.04
Classic sacrificial dilemmas	1–7	5.19	1.36
Greater good dilemmas	1–7	2.34	1.09
Psychopathy	1–4	1.76	.47
Empathic concern	1–5	4.02	.80
Need for cognition	1–5	3.39	.71
Hypothetical donation	0–100	31.56	25.59
Environmental protection	1–7	5.87	1.16
Religiosity	1–5	2.68	1.27
Economic conservatism	1–7	3.77	1.77
Religiosity	1–5	2.68	1.27

## Footnotes

1 Some nonutilitarian moral views, including Kantianism and some forms of Christian and Buddhist ethics, are also radically impartial (e.g., Kant tells us to give equal respect to all rational beings) but that impartiality is not expressed in the goal of maximizing the well-being of all.

2 For ease of presentation, we distinguished here the *degree* of the sacrifices we make to aid others and for *whose sake* we make these sacrifices—specifically, whether in doing so we treat everyone’s well-being as equally important. This could give the impression that impartial beneficence consists of how beneficent we are (degree of sacrifice) and how impartial we are (whether or not we prioritize certain others). However, from a utilitarian standpoint these are not conceptually distinct: prioritizing the self and prioritizing family, friends, or compatriots, are both forms of partiality that utilitarianism rejects (unless such differential treatment can be justified in impartial terms—e.g., if we can generate more utility by taking care of those we know well). Moreover, impartial beneficence needn’t require any sacrifice—as, for example, when one has to choose whether to prevent harm to a family member versus a greater number of strangers. Still, while complete impartiality involves giving equal importance to the good of all individuals (including oneself), departures from such impartiality can go both in the direction of greater prioritisation of self and greater prioritisation of those close to us.

3 Notice, however, that we defined ‘instrumental harm’ as harm that aims to bring about a greater good. Throwing someone onto the track of a train just for fun is thus *not* a case of instrumental harm in our sense. Does this mean that instrumental harm inherently involves an element of impartial beneficence, and is therefore an overlapping construct? Endorsing instrumental harm involves an element of *beneficence*, that is, acting in ways that benefit (or prevent harm to) others and, indeed, in a way that is responsive to the number of lives saved. But such an act typically involves no self-sacrifice nor need it express any kind of impartiality—in typical sacrificial dilemmas, both the person to be sacrificed and those who can be saved are strangers. Notice also that beneficence in this weak sense is a mundane moral notion that is endorsed by virtually all moral views, including stringent deontological theories.

4 At the theoretical level, it is possible to understand giving moral weight to different forms of partiality as themselves reflecting a set of deontological rules. But in the psychological context it is more illuminating to treat this dimension separately.

5 Conway and Gawronski (2013) label a reluctance to endorse directly harming others a ‘deontological inclination’ and giving greater weight to saving more lives a ‘utilitarian inclination.’ But as one of us has argued (Kahane, 2015), this opposition can also be understood as one between a duty not to harm (nonmaleficence) and a duty to rescue/aid (broadly falling within the duty of beneficence). Understood in this way, this opposition really operates *within* a deontological framework (Ross, 2002 system of *prima facie* duties is a classic example of such a framework). As discussed above, what is distinctive of utilitarianism isn’t that it recognizes a duty of beneficence but that it (a) interprets it in a radically impartial and maximizing way, and (b) regards it as the whole of morality. Notice that to approach sacrificial dilemmas (and instrumental harm more generally) in a thoroughly utilitarian manner, it is not enough to give greater weight to beneficence (i.e., to saving five lives); one must also thoroughly reject any deontological constraint on directly harming others—that is, one must *also* have no deontological inclination whatsoever, in Gawronski and Conway’s terms. It is enough that the number of lives saved counts for more; for a utilitarian, it is the *only* thing that counts (Kahane, 2015).

6 We also asked the experts to indicate “How much do you personally agree with this statement?” (1 = *not at all*; 5 = *very much*). These data were not used in the present study.

7 Although the OUS scores correlated well with an explicit statement of utilitarianism, it is also worth highlighting at this junction that we would not recommend using such an explicit statement of utilitarianism as an independent measure of utilitarian tendencies. First, the explicit description does not completely distinguish between the positive and negative components of utilitarianism. Second, and relatedly, although OUS scores correlated well with the explicit statement, this was far from a perfect correlation, and OUS scores often had stronger correlations with the other measures than did the explicit statement. For example, as reported below, scores on the OUS-IB had a much stronger correlation with perceptions of wrongness on the greater good dilemmas from Kahane et al. 2015; discussed below: *r* = .50, *p* < .001), whereas the correlation with the explicit statement of utilitarianism was substantially weaker, *r* = .27, *p* < .001.

8 Instrumental Harm was also independent from other aspects of the ‘negative’ dimension of utilitarianism, such as the rejection of absolutist and traditional morality captured by the third factor that emerged in the scale construction process, a factor that we ultimately did not include in the final scale.

9 Fully, impartial beneficence regards the well-being of all morally relevant subjects as equally important. Exactly which beings fall within the scope of such concern will depend on further questions about well-being, the relationship between well-being and sentience, and which living things possess sentience in the relevant sense (a utilitarian may give equal consideration to the well-being of all sentient beings but hold that, e.g., fish are not sentient in the relevant sense).

10 Impartial beneficence and instrumental harm are logically linked *within* a utilitarian framework: classical utilitarianism tells us that impartial beneficence is the *whole* of morality, which entails that there are no intrinsic constraints on the means we can use to promote everyone’s good. That is to say, instrumental harm is derived from the more fundamental idea of impartial beneficence, when the latter is used to define moral rightness. However, it does *not* follow that the two must go together as a matter of logic. We can regard Impartial Beneficence not as the whole of morality but as one moral principle among others, a principle that can be outweighed or constrained, for example, by restrictions on what kind of harms are morally permitted. Conversely, acceptance of instrumental harm in no way mandates endorsement of impartial beneficence—in the same way that a permissive attitude to ‘white lies’ in no way commits us to accepting duties to aid distant strangers (though instrumental harm does assume a degree of *beneficence*—i.e., giving some weight to the number of lives one can save). So the strong association between impartial beneficence and instrumental harm within expert moral philosophers cannot be fully explained simply as being due to a search for consistency. It may also reflect the fact that both impartial beneficence and instrumental harm are historically associated with utilitarianism, a view that features prominently in introductory courses in ethics.

11 Another distinctive feature of classical utilitarianism that the OUS does not measure directly is its rejection of a moral distinction between acts and omissions. One of the items of the Impartial Beneficence subscale is concerned with rejecting such a distinction in the context of aid, but impartial beneficence is conceptually distinct from the rejection of the act/omissions distinction and the relationship between the two requires further investigation. Such an investigation would also clarify the relation between OUS scores and omission bias, a nonutilitarian disposition that has been studied extensively (see Baron & Ritov, 1994; Ritov & Baron, 1990).
