# Correction to Kahane et al. (2018)

**DOI:** 10.1037/rev0000112

**Published:** 2018-03

In the article “Beyond Sacrificial Harm: A Two-Dimensional Model of Utilitarian Psychology” by Guy Kahane, Jim A. C. Everett, Brian D. Earp, Lucius Caviola, Nadira S. Faber, Molly J. Crockett, and Julian Savulescu (Psychological Review, 2018, Vol. 125, No. 2, pp. 131–164. http://dx.doi.org/10.1037/rev0000093), the copyright attribution was incorrectly listed, and the Creative Commons CC-BY license disclaimer was incorrectly omitted from the author note. The correct copyright is “© 2017 The Author(s)” and the omitted disclaimer is below. All versions of this article have been corrected. 

“This article has been published under the terms of the Creative Commons Attribution License (http://creativecommons.org/licenses/by/3.0/), which permits unrestricted use, distribution, and reproduction in any medium, provided the original author and source are credited. Copyright for this article is retained by the author(s). Author(s) grant(s) the American Psychological Association the exclusive right to publish the article and identify itself as the original publisher.”

