# Supplementary material for: Beyond Sacrificial Harm: A Two-Dimensional Model of Utilitarian Psychology
Source: Psychol Rev. 2017 Dec 21;125(2):131–64. doi: 10.1037/rev0000093 (PMC5900580; doi:10.1037/rev0000093)
Supplement: Supplementary file 1 [file z2q-2521_REV-2016-0507_SUPPL.zip › z2q999172521so1.docx]

**Supplemental Materials**

**Beyond Sacrificial Harm: A Two-Dimensional Model of Utilitarian Psychology**

**by G. Kahane et al., 2017, *Psychological Review***

[**http://dx.doi.org/10.1037/rev0000093**](http://dx.doi.org/10.1037/rev0000093)

# Supplementary Introduction

## Existing Scales

Several scales purporting to measure utilitarian (or, more broadly, “consequentialist”) tendencies have already been developed. However, these suffer from broadly the same limitations as the sacrificial dilemmas we discussed above, given that they similarly focus on negative willingness to break moral rules to bring about a greater good (with a particular emphasis on breaking rules relating to harming).

Consider, for example, the Consequentialist Thinking Scale (Piazza & Sousa, 2013). This scale consists of 14 questions encompassing moral issues such as killing, stealing, and incest. Participants are asked “Which of the following statements best characterizes your position on (e.g., stealing)?” They are then given the choice of one of three options: a statement indicating a deontological response (“It is never morally permissible to steal”); a statement indicating a weak consequentialist response (“If stealing will produce greater good than bad consequences, then it is morally permissible to steal”); and a statement indicating a strong consequentialist response (“If stealing will produce greater good than bad consequences, then it is morally obligatory to steal”). One strength of this scale is that it focuses on practical ethical decisions rather than abstract ethical theories or farfetched scenarios: When faced with difficult moral choices concerning issues like abortion and torture, do people make decisions more along consequentialist or deontological lines? However, this scale still only captures what might be called the “negative” aspect of utilitarianism—the way it allows us to break moral rules to bring about a greater good (Kahane et al., 2015)—yet still ignores utilitarianism’s positive impartial ideal and the practical contexts in which it is manifested (e.g. situations that pit self-interest or concern for those close to us against the well-being of distant strangers).

Another limit of this scale is that it classifies the view that it is *permissible* to break a moral rule to bring about a greater good as a consequentialist response. However, it is merely a commonsensical view that we may be permitted to break some moral rules if this will, for example, prevent a very great harm—or indeed, that we are sometimes required to do so if the alternative is catastrophic. But what is distinctive of utilitarian (and consequentialist views more generally) is that they *require* us, without exception, to break rules whenever this would lead to a somewhat better outcome. Although extreme (and counterintuitive) deontological views, such as Kant’s, forbid us from breaking certain moral rules no matter how severe the consequences (e.g., Kant would say we must not lie to the Nazi at our door asking if we are hiding any Jews), most non-utilitarian views aren’t absolutist. We should not identify deontology with extreme absolutism, or its rejection with consequentialism.

More promising with regard to measuring utilitarianism is Robinson’s unpublished Consequentialist Scale (Robinson, 2012). This consists of two subscales: a 5-item Deontology subscale measuring the extent to which individuals hold deontological moral beliefs (defined as the idea that “some rules should never be broken, regardless of the consequences that result,” p. 6); and a 5-item Utilitarianism subscale, measuring the extent to which individuals hold Utilitarian moral beliefs (defined as the idea that “the only morally relevant dimension to judge an action is whether or not it resulted in a net positive outcome,” p. 6). For each of the 10 items (e.g., “If rules and laws do not maximize happiness for people they should be ignored”), participants rated how much they agreed or disagreed on a 1-5 scale. One advantage of this scale is that all 5 of the utilitarianism subscale items reference the overall maximization of happiness—a critical feature of utilitarianism. And because it consists of only 5, fairly short items, it does not require substantial time from, nor place a heavy intellectual demand on, participants.

One limit of this scale, however, is that most of the items in the utilitarianism subscale are extremely similar: for example, “Rules and laws should only be followed when they maximize happiness” vs. “Rules and laws are irrelevant; whether an action produces happiness is all that matters when deciding how to act” vs. “If rules and laws do not maximize happiness for people they should be ignored”. These items also still focus on the issue of rule-breaking. And while the explicit reference to the maximization of happiness is important, this aim can be understood in more or less impartial ways (the happiness in question might be taken to be only that of members of one’s own society). Participants could therefore endorse such an item without believing that they have the slightest obligation to help people in need in developing countries, let alone feeling concerned about the plight of factory farmed animals. In contrast to the items in the Piazza & Souza scale, then, which focus on concrete practical issues, the items on Robinson’s scale might be too abstract.

# Supplementary Method

## Initial Item Generation

An initial pool of items was generated through a comprehensive survey of the existing literature on utilitarianism. Given that one of the most important motivations for this research was to develop a philosophically sophisticated measurement tool, we paid special attention not only to the best empirical studies from psychology bearing on the subject but also to influential theoretical work from within the philosophical tradition.

In creating the initial pool of items, several considerations were taken into account. First, we judged it necessary to include both items that tapped into the abstract tenets of utilitarianism and items that bore on real-world moral judgments that track utilitarian thinking. Second, it was essential to include items that captured both the positive and negative components of utilitarianism: namely, that the right act is the one that impartially maximizes the greater good (positive component) and that this maximization is all there is to morality such that deontological rules and constraints must be rejected when they stand in the way of achieving this goal (negative component). To that end, we cast a wide net in our initial search of the literature so as not to miss any promising items inadvertently.

In practical terms, we used both a “bottom-up” and a “top-down” approach to identifying relevant items. The “bottom-up” approach involved compiling studies from the existing empirical literature purporting to measure utilitarian judgments and extracting relevant source citations from their references sections in a systematic fashion (e.g., Piazza & Sousa, 2013; Robinson, 2012; Greene et al., 2001; Moore, Clark, & Kane, 2008). This resulted in well over 200 items, mostly in the form of vignettes or short statements describing moral dilemmas. An initial review of these items revealed considerable redundancy in terms of theoretical content, with the majority of cases clustering around variants of the well-known “push” and “switch” dilemmas (Foot, 1967; Greene et al., 2001; Thomson, 1985). We therefore deemed it necessary to perform a “top-down” analysis, as well, to ensure a more robust theoretical foundation. For this analysis, we drew on philosophical literature and the expertise of professional moral philosophers—including members of the present research team—to identify the most important theoretical tenets of utilitarianism, taking into account differences between the major schools of thought, which we then used as a guide for a more targeted search through the philosophical literature.

For this search, we reviewed both the classical and more recent literature on utilitarianism. Classical statements of the theory included those by Bentham (1789/1983), Mill (1863), and Sidgwick (1901); important recent contributions included work by, for example, Smart and Williams (1973), along with critiques (e.g., Rawls, 1971) and defenses (e.g., Kagan, 1989) of utilitarianism, as well as influential further developments of the theory (e.g., Parfit, 1984). We also took care to include works focusing on the practical implications of utilitarianism (e.g., Singer, 1993).

In reviewing these works, our aim was to identify or, as necessary, formulate, items whose endorsement or rejection would strongly distinguish utilitarian from non-utilitarian views. To this end, we included: (a) abstract statements of utilitarian belief (e.g., “From a moral perspective, people should care about the well-being of all human beings on the planet equally; they should not favor the well-being of people who are especially close to them either physically or emotionally”), (b) anti-utilitarian views (e.g. “There are some things that are simply right or wrong, no matter what the consequences”), (c) items reflecting the application of utilitarianism to concrete contexts (e.g. “It is morally wrong to keep money that one doesn’t really need if one can donate it to causes that provide effective help to those who will benefit a great deal”), and (d) items briefly stating seminal examples or illustrations used by both critics and defenders of utilitarianism ( e.g. "If letting an innocent person go free in some particular instance would certainly cause riots—riots that would lead to a serious loss of life—then it is OK to send this innocent person to jail", based on McCloskey, 1965). Dozens of further items were added to our initial set as a result of this top-down approach.

The next step was to filter out major redundancies between items, irrelevant items, and poorly-worded or confusing items, which was done on the basis of an item-by-item assessment of the initial set performed by the present researchers, with discussion as needed to resolve competing assessments. This resulted in a smaller pool of 93 items that were then edited for theoretical clarity and ease of understanding.

## Supplementary Construct Validity

### Robinson’s Consequentialist Scale

We compared responses on the OUS with those on Robinson’s (2012) unpublished 10-item Consequentialism Scale, which we judged as being more similar to our criteria in its aims and format than other, published scales (Piazza & Sousa, 2013). Robinson’s Consequentialism Scale consists of two subscales: a 5-item Deontology subscale measuring the extent to which individuals hold deontological moral beliefs (defined as the idea that “some rules should never be broken, regardless of the consequences that result” p.6); and a 5-item Utilitarianism subscale, measuring the extent to which individuals hold Utilitarian moral beliefs (defined as the idea that “the only morally relevant dimension to judge an action is whether or not it resulted in a net positive outcome”). For each of the 10 items (e.g. “If rules and laws do not maximize happiness for people they should be ignored”), participants rated how much they agreed or disagreed on a 1-5 scale.

As expected, overall scores on the OUS were positively associated with scores on Robinson’s Utilitarianism subscale (*r* = .26, *p* < .001), and this was the same for the OUS-IB (*r* = .25, *p* < .001) and the OUS-IH (*r* = .14, *p* = .02). Looking at the relationship between OUS scores and responses to Robinson’s Deontology subscale revealed a more nuanced pattern. Interestingly, overall OUS scores were not related to scores on the Deontology subscale (*r* = .09, *p* = .12)—but this was because the two OUS subscales were associated with Robinson’s deontology scores in different directions. Although there was a negative but non-significant correlation with the OUS-IH *(r* = −.06, *p* = .30), there was a significant *positive* relationship with the OUS Impartial Beneficence subscale such that people who scored higher on Robinson’s Deontology subscale actually also reported greater concern for the impartial maximization of welfare (*r* = .18, *p* = .003).

Although this result may seem surprising, it confirms the suggestion by Kahane et al. (2015) that the positive and negative dimensions of utilitarianism may be not only psychologically independent but even potentially opposed. Someone may be willing to harm others to bring about a better outcome without being impartially concerned with the greater good of all. Conversely, someone may endorse impartial beneficence while still holding that certain harmful means of achieving this impartial aim are not morally permissible. This possibility is obscured if we treat utilitarianism as a unitary psychological construct.

## Personality and Individual Differences

### Need for Cognition

It has been argued that utilitarian decision-making is uniquely based on effortful conscious reasoning (as opposed to immediate emotional responses) and that utilitarian inclinations are uniquely associated with a need for cognition (Conway & Gawronski, 2013); the motivational tendency to engage in effortful thinking (Cacioppo, Petty, & Kao, 1984). Indeed, the tendency for an individual to engage in and enjoy thinking seems intuitively related to utilitarianism to the extent that utilitarian decision-making involves the rational weighing of different consequences and the rejection of simpler intuitive solutions to moral problems. It has also often been claimed that greater reliance on reason was the source of the historical emergence of utilitarianism, and generally of more impartial and inclusive moral views (Pinker, 2011; Singer, 2011). The extent to which need for cognition might have divergent relations with the twin factors of impartial beneficence and instrumental harm, though, remains unclear. To explore this with the OUS, we had participants complete the 18-item Need for Cognition scale (Cacioppo et al., 1984), in which participants rate how characteristic or uncharacteristic certain statements are of them, such as “I would prefer complex to simple problems” (1 *= extremely uncharacteristic of me;* 7 *= extremely characteristic of me*).

In contrast to some previous research, there was no relationship between need for cognition and OUS scores: not overall (*r* =.02, *p* = .73) or the OUS-IB (*r* =.06, *p* = .35) or OUS-IH separately (*r* =-.03, *p* = .57). And interestingly, there was no association between need for cognition and other relevant measures, such as Robinson’s utilitarianism scale (*r* = -.09, *p* = .13) or sacrificial moral dilemmas (*r* = -.04, *p* = .46).

| Table 8b. *Correlations Between the OUS And Related Individual Differences Measures* | | | | |
| --- | --- | --- | --- | --- |
|  | 1 | 2 | 3 |  |
| 1. Overall Oxford Utilitarianism Scale (OUS) | - |  |  |  |
| 2. Impartial Beneficence Sub-Scale (OUS-IB) | .81^**^ | - |  |  |
| 3. Instrumental Harm Sub-Scale (OUS-IH) | .70^**^ | .14^*^ | - |  |
| 4. Anti-Traditional Morality | -.14^*^ | -.08 | -.15^*^ |  |
| 5. Empathic Concern | .14^*^ | .33^**^ | -.16^**^ |  |
| 6. Psychopathy | .11 | -.09 | .30^**^ |  |
| 7. Need for Cognition | .02 | .06 | -.03 |  |
| 8. Identification with all of Humanity | .13^**^ | .33^**^ | -.19** |  |
| *Note.* * *p* <.01, ** *p* < .005 | | | | |

| Table 9b. *Correlations Between the OUS And Ideology* | | | | |
| --- | --- | --- | --- | --- |
|  | 1 | 2 | 3 | 4 |
| 1. Overall Oxford Utilitarianism Scale (OUS) | - |  |  |  |
| 2. Impartial Beneficence Sub-Scale (OUS-IB) | .81^**^ | - |  |  |
| 3. Instrumental Harm Sub-Scale (OUS-IH) | .70^**^ | .14^*^ | - |  |
| 4. Anti-Traditional Morality | -.14^*^ | -.08 | -.15^*^ | - |
| 5. Hypothetical Donation | .31^**^ | .40^**^ | .03 | -.05 |
| 6. Purity Dilemma | .02 | .05 | -.04 | -.38^**^ |
| 7. Environmental Protection | -.03 | .14^*^ | -.21^**^ | -.05 |
| *Note.* * *p* <.01, ** *p* < .005 | | | | |

| Table 10b. *Correlations Between the OUS And Ideology* | | | | |
| --- | --- | --- | --- | --- |
|  | 1 | 2 | 3 | 4 |
| 1. Overall Oxford Utilitarianism Scale (OUS) | - |  |  |  |
| 2. Impartial Beneficence Sub-Scale (OUS-IB) | .81^**^ | - |  |  |
| 3. Instrumental Harm Sub-Scale (OUS-IH) | .70^**^ | .14^*^ | - |  |
| 4. Anti-Traditional Morality | -.14^*^ | -.08 | -.15^*^ | - |
| 5. Economic Conservatism | .02 | -.12 | .18^**^ | -.31^**^ |
| 6. Social Conservatism | .06 | -.06 | .18^**^ | -.41^**^ |
| 7. Religiosity | .15^*^ | .15^*^ | .06 | -.48^**^ |
| *Note.* * *p* <.01, ** *p* < .005 | | | | |

| Table 11b. *Ms And Sds For Measures in Study 2* | | | |
| --- | --- | --- | --- |
| Measure | Scale Rating | M | SD |
| Overall Oxford Utilitarianism Scale (OUS) | 1-7 | 3.50 | 0.92 |
| Impartial Beneficence Sub-Scale (OUS-IB) | 1-7 | 3.65 | 1.20 |
| Instrumental Harm Sub-Scale (OUS-IH) | 1-7 | 3.31 | 1.22 |
| Anti-Traditional Morality | 1-7 | 3.46 | 1.21 |
| Explicit Utilitarianism | 1-5 | 2.99 | 1.04 |
| Robinson’s Deontology Sub-Scale | 1-5 | 3.38 | 0.75 |
| Robinson’s Consequentialism Sub-Scale | 1-5 | 2.29 | 0.77 |
| Classic Sacrificial Dilemmas | 1-7 | 5.19 | 1.36 |
| Greater Good Dilemmas | 1-7 | 2.34 | 1.09 |
| Empathic Concern | 1-5 | 4.02 | 0.80 |
| Psychopathy | 1-4 | 1.76 | 0.47 |
| Need for Cognition | 1-5 | 3.39 | 0.71 |
| Environmental Protection | 1-7 | 5.87 | 1.16 |
| Hypothetical Donation | 0-100 | 31.56 | 25.59 |
| Economic Conservatism | 1-7 | 3.77 | 1.77 |
| Social Conservatism | 1-7 | 3.21 | 1.78 |
| Religiosity | 1-5 | 2.68 | 1.27 |

# 
